# Declining carbohydrate solubilization with increasing solids loading during fermentation of cellulosic feedstocks by *Clostridium thermocellum*: documentation and diagnostic tests

**DOI:** 10.1186/s13068-022-02110-4

**Published:** 2022-02-05

**Authors:** Matthew R. Kubis, Evert K. Holwerda, Lee R. Lynd

**Affiliations:** 1 Thayer School of Engineering, Dartmouth College, 14 Engineering Drive, Hanover, NH 03755 USA; 2 The Center for Bioenergy Innovation, Oak Ridge National Laboratory, Oak Ridge, TN 37831 USA

**Keywords:** Corn stover, Switchgrass, High solid loading, Biomass deconstruction, Lignocellulose, Cellulose, Hemicellulose, Biofuels, *Clostridium thermocellum*, *Thermoanaerobacterium thermosaccharolyticum*, Coculture

## Abstract

**Background:**

For economically viable 2nd generation biofuels, processing of high solid lignocellulosic substrate concentrations is a necessity. The cellulolytic thermophilic anaerobe *Clostridium thermocellum* is one of the most effective biocatalysts for solubilization of carbohydrate harbored in lignocellulose. This study aims to document the solubilization performance of *Clostridium thermocellum* at increasing solids concentrations for two lignocellulosic feedstocks, corn stover and switchgrass, and explore potential effectors of solubilization performance.

**Results:**

Monocultures of *Clostridium thermocellum* demonstrated high levels of carbohydrate solubilization for both unpretreated corn stover and switchgrass. However, fractional carbohydrate solubilization decreases with increasing solid loadings. Fermentation of model insoluble substrate (cellulose) in the presence of high solids lignocellulosic spent broth is temporarily affected but not model soluble substrate (cellobiose) fermentations. Mid-fermentation addition of cells (*C. thermocellum*) or model substrates did not significantly enhance overall corn stover solubilization loaded at 80 g/L, however cultures utilized the model substrates in the presence of high concentrations of corn stover. An increase in corn stover solubilization was observed when water was added, effectively diluting the solids concentration mid-fermentation. Introduction of a hemicellulose-utilizing coculture partner, *Thermoanaerobacterium thermosaccharolyticum,* increased the fractional carbohydrate solubilization at both high and low solid loadings. Residual solubilized carbohydrates diminished significantly in the presence of *T. thermosaccharolyticum* compared to monocultures of *C. thermocellum,* yet a small fraction of solubilized oligosaccharides of both C_5_ and C_6_ sugars remained unutilized.

**Conclusion:**

Diminishing fractional carbohydrate solubilization with increasing substrate loading was observed for *C. thermocellum*-mediated solubilization and fermentation of unpretreated lignocellulose feedstocks. Results of experiments involving spent broth addition do not support a major role for inhibitors present in the liquid phase. Mid-fermentation addition experiments confirm that *C. thermocellum* and its enzymes remain capable of converting model substrates during the middle of high solids lignocellulose fermentation. An increase in fractional carbohydrate solubilization was made possible by (1) mid-fermentation solid loading dilutions and (2) coculturing *C. thermocellum* with *T. thermosaccharolyticum*, which ferments solubilized hemicellulose. Incomplete utilization of solubilized carbohydrates suggests that a small fraction of the carbohydrates is unaffected by the extracellular carbohydrate-active enzymes present in the culture.

**Supplementary Information:**

The online version contains supplementary material available at 10.1186/s13068-022-02110-4.

## Background

Conversion of lignocellulose feedstocks has attracted global interest as a sustainable source of transportation fuels. Biologically mediated events in lignocellulose conversion include production of carbohydrate-active enzymes (CAZymes), enzymatically mediated carbohydrate solubilization, and fermentation of soluble sugars [1]. Plants have evolved to be resistant to biological attack, and overcoming this recalcitrance is responsible for the high cost of current conversion technology [2]. The most widely studied strategy for solubilizing the carbohydrate fraction of cellulosic biomass involves thermochemical pretreatment and added enzymes produced by aerobic fungi [3–5]. Alternatively, some thermophilic anaerobes are naturally capable of producing cellulases and other CAZymes and then fermenting the solubilized carbohydrates to a desired product in a one-step approach called consolidated bioprocessing (CBP) [6]. Mechanical disruption during fermentation (cotreatment) has also been proposed as an alternative to thermochemical pretreatment to augment biologically mediated deconstruction (C-CBP), but is not the focus of this study [7–9].

*Clostridium thermocellum (Ruminiclostridium thermocellum, Hungateiclostridium thermocellum, Acetivibrio thermocellus)*, a cellulolytic and thermophilic anaerobic bacterium, is the most efficient microorganism at lignocellulose solubilization known [7, 10], and is thus a promising candidate for CBP. Although the biomass deconstruction apparatus of *C. thermocellum* equally solubilizes pentose-rich hemicellulose as well as cellulose [7], wild-type strains do not ferment pentose sugars [11–13]. As a result, xylo-oligomers accumulate when lignocellulose is fermented by *C. thermocellum* monocultures. Pentose sugars and oligomers originating from hemicellulose have a deleterious effect on lignocellulose deconstruction by fungal cellulase preparations [14–17]. There have been reports of possible effects of soluble hemicellulose on cultures of *C. thermocellum* and cell-free cellulase preparations on switchgrass [18, 19] and corn fiber [20].

In addition to potentially affecting cellulolytic activity, failure to utilize C_5_ sugars decreases the product yield per unit biomass. To avoid these undesired effects, defined cocultures of *C. thermocellum* with a compatible hemicellulose-fermenting strain have been studied, and identifying synergistic coculture partners is important in the context of biofuel production by CBP [21]. On both microcrystalline cellulose and lignocellulose, Ng et al. reported that cocultures with the hemicellulose-utilizing *Clostridium thermohydrosulfuricum (renamed as Thermoanaerobacter thermohydrosulfuricus)* enhanced substrate consumption and ethanol yield [22]. Saddler and Chan had similar observations for *C. thermocellum* NRCC 2688 cocultured with *Clostridium thermosaccharolyticum* on pretreated wheat straw [23]. Coculture studies with *Thermoanaerobacterium* spp. on microcrystalline cellulose demonstrated increased product formation on crystalline cellulose in comparison to *C. thermocellum* monocultures [24–26]. Recently, Froese et al. (2018) reported 2 g/L wheat straw cocultures with either *Ruminiclostridium stercorarium* or *Thermoanaerobacter thermohydrosulfuricus* improved carbohydrate solubilization by 30% relative to the monoculture as determined by measuring end products and soluble oligosaccharides [27]. Beri et al. showed cocultures with hemicellulose-utilizing *Herbinix spp.* and *Thermoanaerobacterium thermosaccharolyticum* on 40 g/L corn fiber resulted in a 1.39-fold increase (67% to 93%) in overall solubilization relative to the monoculture. In another study, Beri et al. described an inhibitory effect relating to a hemicellulose component of corn fiber, glucuronoarabinoxylan (GAX) [20] Supplementation of enzymes capable of disrupting the GAX-linkages alleviated inhibition and improved carbohydrate solubilization from 33 to 63% for 40 g/L corn fiber solids loading [28].

High loadings of lignocellulosic feedstocks are required for industrial feasibility in order to avoid high costs for product recovery (e.g., steam for distillation) as well as high capital costs for bioreactors [2, 3]. For lignocellulose solubilization mediated by fungal cellulase preparations, a decrease in solubilization has been observed with increasing solid loadings [29–33]. In these systems, mass transfer and free water limitations arise as solid loadings approach 15–20% dry matter [30, 33], yet several other causative factors have also been reported including, but not limited to, the presence and/or accumulation of ethanol [34, 35], glucose [34, 36], cellobiose [34, 36], xylan [14, 37–40], xylose [15], xylo-oligomers [16, 17, 38], lignin [35, 41], and inhibitors related to thermochemical pretreatment [35, 42]. For mixed anaerobic consortia cultivated during anaerobic digestion (AD), it has been observed that no significant differences in the specific methane yield occur between 1 and 15% total solids with a decrease eventually observed at 20–30% total solids [43–48]. In these studies, mass transfer limitations due to lack of free water and/or organic overfeeding have been cited as possible explanations [44, 45].

For defined cultures of thermophilic anaerobes aimed at lignocellulose solubilization, most studies have targeted documenting and understanding capability at solids loadings ≤ 20 g/L. At carbohydrate loadings up to 120 g/L, *C. thermocellum* cultures solubilize 80–93% of the cellulose present in Avicel® PH105, a model microcrystalline cellulose substrate containing negligible amounts of both lignin and hemicellulose [49–51]. However, declining solubilization with increasing loading has been observed for such cultures when fermenting unpretreated lignocellulose. Verbeke et al. [52] observed a 1.72-fold decline in solubilization extent between 50 and 10 g/L on mid-season switchgrass that could not be solely explained by either recalcitrance or inhibition by fermentation products [52], and Shao et al. [19] also reported diminishing solubilization at increasing loadings of mid-season switchgrass [19]. Both authors described a deleterious effect to lignocellulose deconstruction in the presence of C_5_ sugars, either in monomeric or oligomeric forms [18]. Similarly for corn fiber, Beri et al. observed declining solubilization (90–67%) between 20 and 40 g/L which was largely overcome by coculturing *C. thermocellum* with a hemicellulose-utilizing thermophile [20]. *Caldicellulosiruptor bescii,* a hyperthermophilic, cellulolytic anaerobe [53], experiences a 1.8-fold decline in carbohydrate solubilization between 5 and 50 g/L switchgrass loadings, and the authors describe overcoming this decline by slowly purging the bioreactor liquid with substrate-free medium [54].

Here we extend the work of Verbeke, Shao and Beri et al. by documenting solubilization as a function of solids concentration by *C. thermocellum* for corn stover and senescent switchgrass with and without *T. thermosaccharolyticum,* and present experiments aimed at evaluating potential causal mechanisms.

## Results

### Fermentation of C. thermocellum with increasing solids loading

**Fig. 1:**
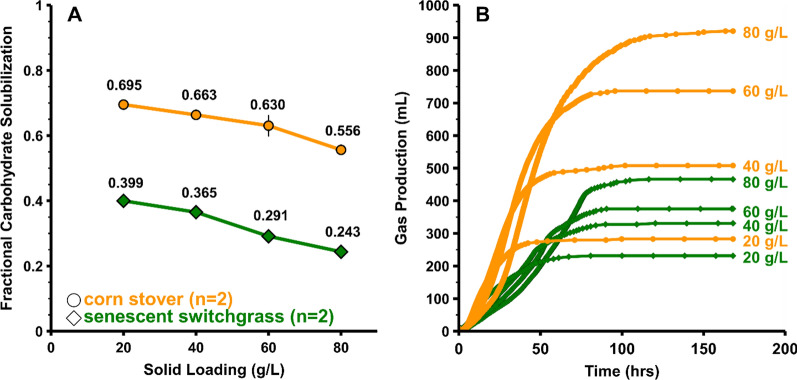
Fermentation of corn stover and senescent switchgrass by monocultures of Clostridium thermocellum at various solid loadings (*n* = 2). **A** Fractional carbohydrate solubilization with error bars representing 1 standard deviation shown (*n* = 2). **B** Representative total gas production (cumulative CO_2_ and H_2_) for one of the duplicate reactors

We aimed to document the impact of solids loading on fractional carbohydrate solubilization (FCS) of corn stover and senescent switchgrass in batch, pH-controlled monocultures of *Clostridium thermocellum* DSM1313 incubated for 7 days at 55 °C. Both of these substrates were milled to 0.5 mm particle size and underwent no pretreatment other than autoclaving. FCS decreases roughly linearly as the initial substrate loading is increased from 20 to 80 g/L (Fig. 1A). Total gas production (accumulative CO_2_ and H_2_) increased with increasing substrate loading and indicated that fermentative activity stopped at about 100–120 h for the highest solid loadings and around 50 h for the lowest solid loadings (Fig. 1B).

As may be observed from Fig. 1, FCS decreased by similar absolute amounts for corn stover (0.139 = 0.695 – 0. 556) and for senescent switchgrass (0.156 = 0.399 – 0.243), corresponding to a 20% decrease for corn stover and a 39% decrease for switchgrass. FCS at each solids loading was higher for corn stover than for switchgrass by a factor of approximately two. The total fraction of carbohydrate per solids was different for corn stover (0.676) and switchgrass (0.725), therefore the carbohydrate loading at equal solid loadings varied slightly for the two feedstocks.

**Fig. 2:**
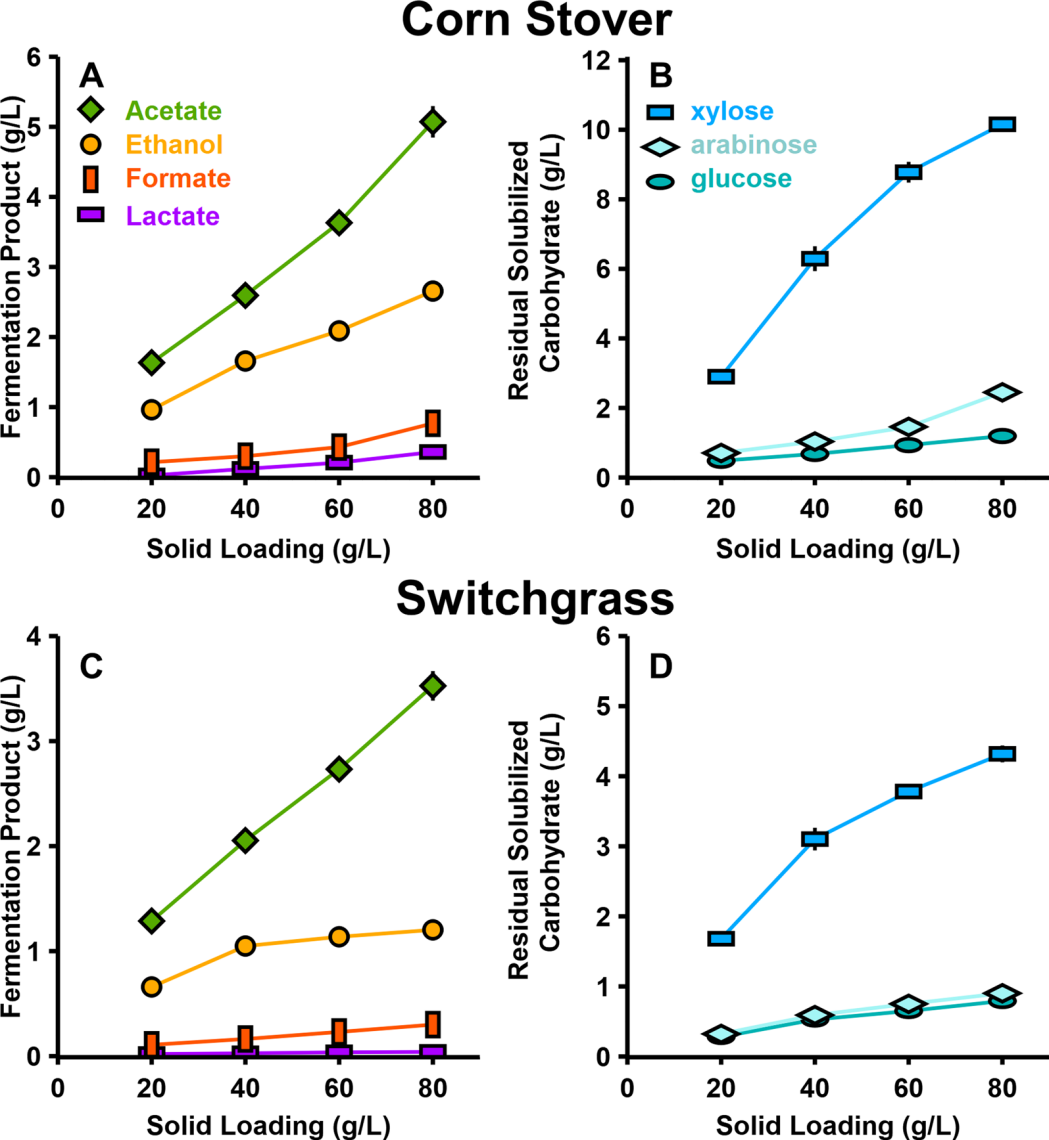
Fermentation products and residual solubilized carbohydrates at various solid loadings for monocultures of Clostridium thermocellum. Panels **A** corn stover and **C** senescent switchgrass fermentation products ethanol, acetate, formate, and lactate. Panels **B** corn stover and **D** senescent switchgrass residual xylose, arabinose, and glucose in the fermentation broth quantified in monomeric form via mild acid hydrolysis of the supernatant. Error bars represent 1 standard deviation for all datapoints in all panels (*n* = 2)

With increasing solid loadings, final concentrations of fermentation products (ethanol, acetate, formate, and lactate shown in Fig. 2, panels A and C) increased. Note that acetate can originate as a product of fermentation or a product of the solubilization process as it is present in the feedstock as acetyl bonds. According to estimates determined by Kumar et al. and Wyman et al., the acetyl content of corn stover and switchgrass is 2.51% and 3.60%, respectively [55, 56]. Based on carbohydrate solubilization, the fraction of acetate that is the result of fermentation would then range between 73.9–78.6% and 74.4–80.1% for corn stover and switchgrass, respectively (Additional file 1: Table S10). Molar ratios of the various fermentative products did not significantly change as solid loadings increased, with the exception of decreasing ethanol production in switchgrass fermentations (Additional file 1: Table S2).

Solubilized and unutilized carbohydrates exist as a mixture of complex oligomers in fermentation broth [28], but were measured in monomeric form after a mild acid hydrolysis step (see Methods), and residual solubilized carbohydrates increased with solid loadings (Fig. 2, panels B and D). While *C. thermocellum* strictly utilizes C_6_ carbon sugars in the form of dimers and homo-oligomers of glucose linked by β-glycosidic bonds [57], a small fraction of the residual unutilized soluble carbohydrates appears to contain glucose. The other sugars present in the unutilized solubilized carbohydrates were xylose and arabinose, which is consistent with the inability of wild-type *C. thermocellum* to utilize C_5_ sugars [11, 12].

### Testing spent broth for inhibitory effects

**Fig. 3:**
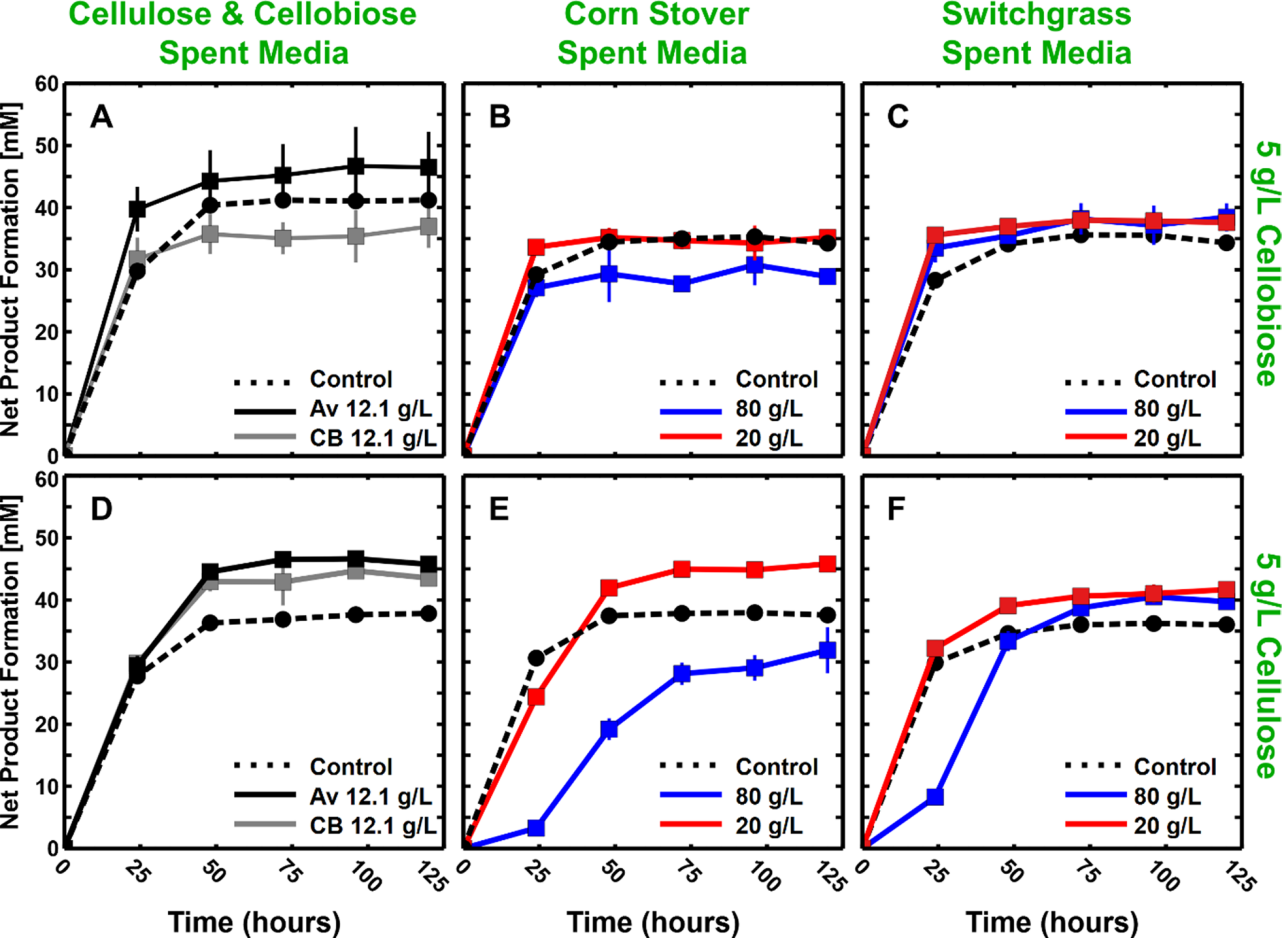
Spent media inhibition tests. The effect of added spent media on net cumulative product formation (acetate + ethanol + formate + lactate in [mM]) during fermentation of 5 g/L cellobiose (panels **A**, **B**, **C**) or cellulose (panels **D**, **E**, **F**) for monocultures of Clostridium thermocellum. Panels B and E show the effect of spent media from corn stover fermentations by C. thermocellum at two different initial solid loadings, and panels C and F show the effect of spent media at two different senescent switchgrass loadings. Panels **A** and **D** show results for addition of spent media from a cellobiose (**A**) and cellulose (**D**) fermentations with carbohydrate utilization equal to that of 80 g/L corn stover solubilization (12.1 g/L cellobiose (CB) or cellulose (Av)). Individual controls (*n* = 1, dashed lines) with water in lieu of spent broth were included for each condition, whereas all other datapoints represent the average of triplicate bottle fermentations (*n* = 3). Error bars represent 1 standard deviation

Spent fermentation broth from the aforementioned 80 (high) and 20 (low) g/L fermentations was collected and examined for inhibitory effects in subsequent bottle fermentations with fresh media and fresh substrate. The spent broth was centrifuged and filtered at 0.2 µm to remove cells and any remaining solids. Additional filtered spent broth was generated by fermenting 12.1 g/L cellobiose or cellulose, corresponding to the amount of glucan solubilized in an 80 g/L corn stover fermentation. The spent media then was aseptically added in 75% volumetric amounts (15-ml) to a 20-mL final volume serum bottle containing fresh media and either 5 g/L microcrystalline cellulose (Avicel PH105) or 5 g/L cellobiose (concentration prior to inoculation) as carbon and energy source (Fig. 3). Bottles with 75% v/v water instead of spent broth served as internal controls. To see if the spent broth had any transient or permanent effects on the solubilization and utilization processes, the net product formation was measured every 24 h for a total of 5 days (product concentration measured minus product concentration present at start of incubation). As shown in Fig. 3, net product formation at the end of bottle incubation was comparable for those with and without spent broth, suggesting the spent broth has limited effects towards the final utilization of model substrates for *C. thermocellum*. However, there was a temporary lag in product formation of up to 50–75 h in the bottles containing cellulose and spent broth from high solid loadings (80 g/L). This delay was not observed for fermentation with cellobiose or at low solid loadings (20 g/L).

### Addition of cells and substrates during fermentation of 80 g/L corn stover

In order to gain diagnostic insights into declining solubilization with increasing substrate loading, model substrates and cells were added 48 h after inoculating duplicate 300-ml batch cultures of *C. thermocellum* in pH-controlled bioreactors with an initial corn stover loading of 80 g/L. Corn stover was chosen as it gave higher solubilization results compared to switchgrass in the previous experiment. Several additions were made including 60-ml solutions of microcrystalline cellulose (Avicel PH105), cellobiose, cellobiose-grown cells concentrated by centrifugation, and water which served as an internal control. Additions of cellobiose and cellulose increased the total carbohydrate loading by 32% (from 54.1 to 71.5 g/L) and were intended to be approximately equal to the observed amount of glucan solubilized in a previous 80 g/L corn stover fermentation. The amount of cellobiose-grown cells added could theoretically represent an increase in cell concentration by 4 g/L, but the overall cell concentration or increase thereof was not measured.

**Fig. 4:**
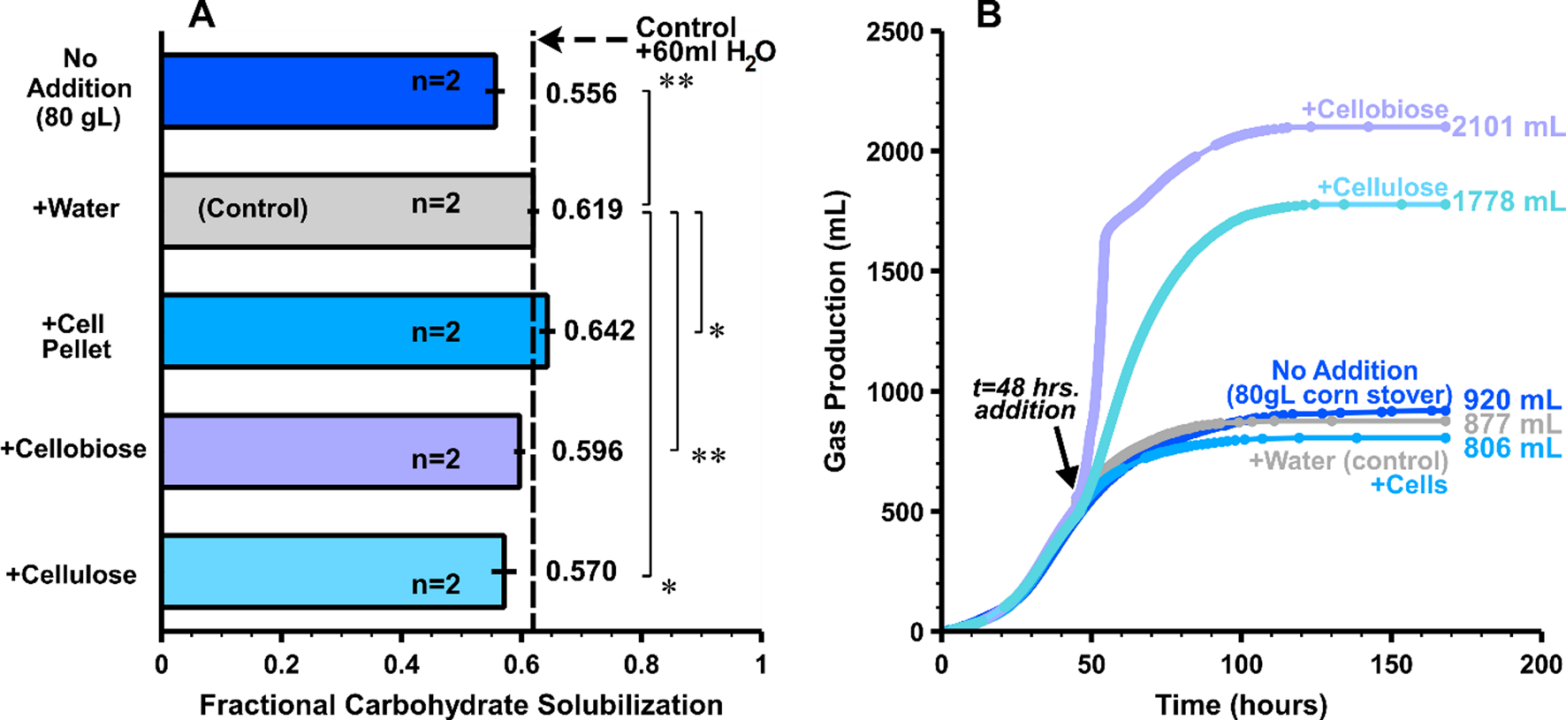
Fermentation addition tests. Panel **A** Fractional carbohydrate solubilization for 80 g/L corn stover with addition of cells (cellobiose-grown and centrifuged cell pellet), cellobiose solution, and suspended Avicel slurry at 48 h after inoculation versus a control (water only) or no addition (*n* = 2). Panel **B** Representative total gas production (cumulative CO_2_ and H_2_) for one of the duplicate reactors. Solubilization bars are averages of duplicate bioreactor runs, error bars represent 1 standard deviation. A single asterisk denotes a significance level of 0.1, and double asterisks denote a significance level of 0.05 between different runs as indicated in the figure. The statistical analyses were performed using t-tests for two-samples assuming unequal variance, and the results are available as supplemental files

As can be seen in Fig. 4, panel A, mid-fermentation addition of cellulose or cellobiose did not enhance solubilization relative to the water control. For the addition of cellulose, fractional carbohydrate solubilization was calculated based on the initial quantity of carbohydrate in the corn stover and the recovered carbohydrate after fermentation, with added cellulose not included in the initial amount of carbohydrate. An immediate increase in gas production accompanied both substrate additions (Fig. 4, panel B), from which we infer that the culture was active and substrate-limited in the absence of added substrate. This is mirrored by an increase in product formation in amounts expected if all added cellobiose and cellulose were utilized (see Additional file 1: Figure S6). Essentially complete fermentation of added substrate is also indicated by low amounts of residual sugars in cultures with and without added substrate (see Additional file 1: Supplemental S5). Extensive utilization of added substrates also suggests that limitation of media components and fermentation product inhibition are not in effect.

Although the highest FCS value was obtained with the mid-fermentation addition of 60 mL concentrated cell suspension, this was not determined to have a significant (p > 0.05) effect on carbohydrate solubilization compared to the water addition control (Fig. 4, panel A), and gas production was very similar to the water addition control (Fig. 4, panel B). The added cell culture proved to be viable as witnessed by normal growth on cellobiose or cellulose in serum bottle incubations inoculated at the same time with the same cell suspension as the addition experiment. We infer from these results that the amount of biocatalyst did not limit solubilization of the carbohydrate present in corn stover at a substrate loading of 80 g/L.

Whereas the fraction of corn stover carbohydrate solubilized was 0.556 ± 0.011 without added cells or substrate, this increased to 0.619 ± 0.005 for the water control. The difference between these values was statistically significant (*p* = 0.020). The addition of water brought the solids loading from 80 g/L to 67 g/L based on initial solids loaded, while the FCS value can be found between 60–80 g/L initial solids loading as shown in Fig. 1.

### Coculture experiments with low and high loadings of corn stover and switchgrass

**Fig. 5:**
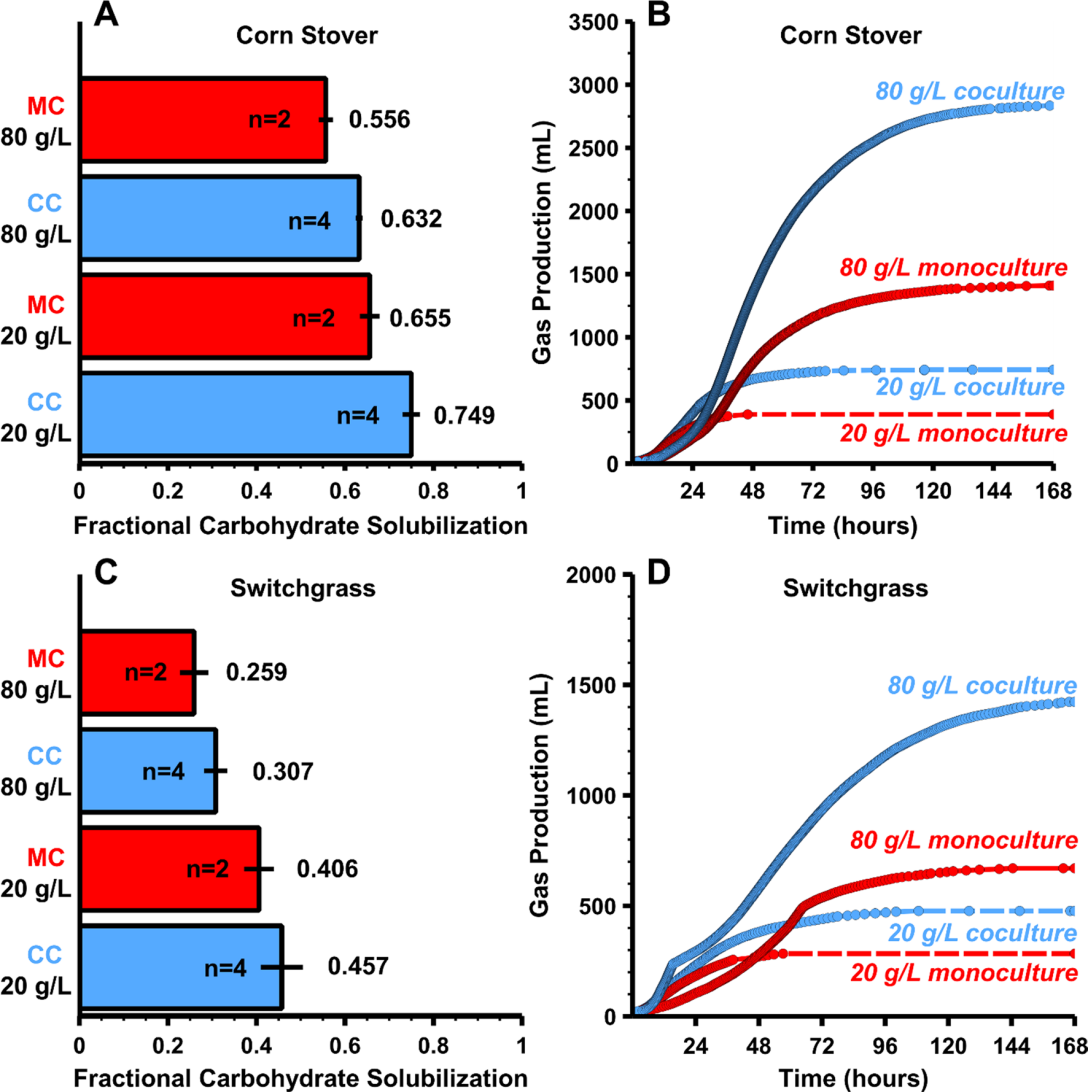
Monocultures and cocultures solubilization and gas production. Panels **A** and **C** Fractional carbohydrate solubilization of 20 g/L and 80 g/L corn stover (**A**) and senescent switchgrass (**C**) by monocultures of Clostridium thermocellum and cocultures of Thermoanaerobacterium saccharolyticum at pH 6.5. Panels **B** and **D** Representative total gas (cumulative CO_2_ and H_2_) production for one of the duplicate reactors of 20 and 80 g/L corn stover (**B**) and senescent switchgrass (**D**) fermentations at pH 6.5. Solubilization bars are averages of duplicate bioreactors runs, and error bars represent 1 standard deviation

We next examined the effect of culturing *C. thermocellum* with a hemicellulose-utilizing coculture partner, *Thermoanaerobacterium saccharolyticum* HG-8 ATCC 31960 for both high (80 g/L) and low (20 g/L) loadings of corn stover and switchgrass. For corn stover fermentations by monocultures and cocultures, FCS data are presented in Fig. 5 panel A and gas production data in panel B. Panels C and D present the same data for switchgrass. The coculture demonstrated higher solubilization (5A and 5C) and gas production (5B and 5D) for both feedstocks at low and high solid loadings. While the coculture showed higher solubilization than the monoculture, it still exhibited diminishing solubilization as with increasing solid loadings.

Two-way ANOVA results indicate that the solids loading effect is significant at p < 0.001 for both substrates, while the coculture effect is significant at p < 0.001 for corn stover and *p* = 0.063 for switchgrass. The net increase in FCS due to the coculture was roughly twice as large for corn stover than for switchgrass, though the relative increases are consistent with the lower fractional solubilization observed for switchgrass monocultures throughout this study. Higher standard deviations were observed for switchgrass than for corn stover, consistent with the lower significance level per ANOVA testing.

**Fig. 6:**
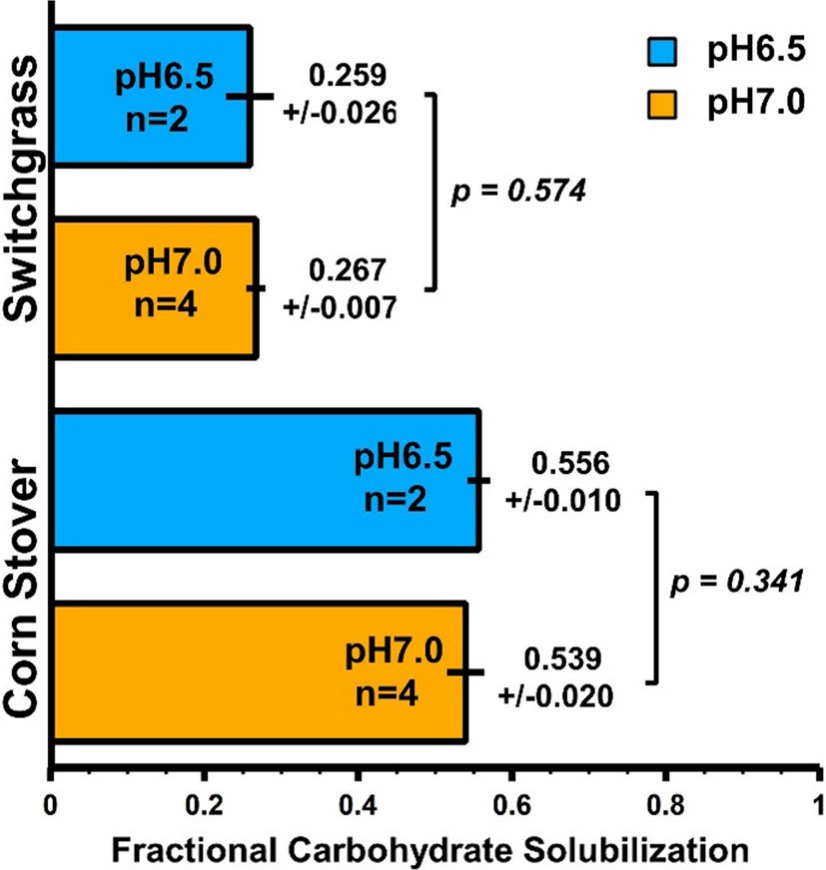
Monoculture solubilization at two pH levels. Fractional carbohydrate solubilization for 7-day Clostridium thermocellum monoculture fermentations of 80 g/L senescent switchgrass and corn stover at pH 6.5 and pH 7.0. The statistical analyses were performed using t-tests for two-samples assuming unequal variance, and the results are available as supplemental file 3

The operating pH during cultivation for coculture experiments was lowered from 7.0 to 6.5 for this set of experiments to better accommodate growth of *T. thermosaccharolyticum* (Additional file 1: Figure S7). For 80 g/L substrate loadings, this was determined to have a non-significant effect on the FCS by the monoculture (Fig. 6).

**Fig. 7:**
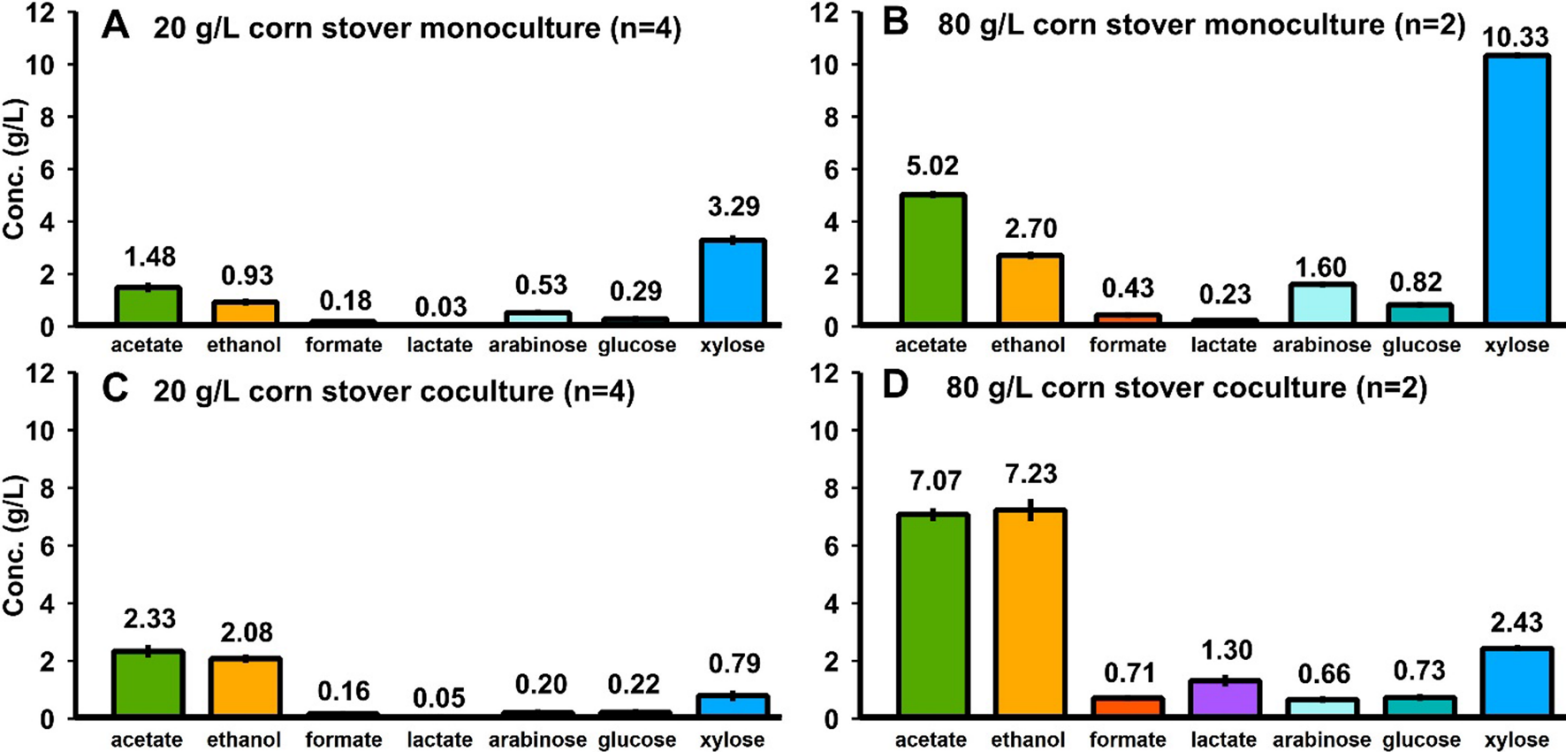
Monocultures and cocultures fermentation products and residual soluble carbohydrate for corn stover. 7-day fermentations of corn stover by monocultures at loadings of 20 g/L (**A**) and 80 g/L (**B**) and cocultures at loadings of 20 g/L (**C**) and 80 g/L (**D**). Residual soluble carbohydrate was quantified in monomeric form after mild acid hydrolysis of the supernatant

**Fig. 8:**
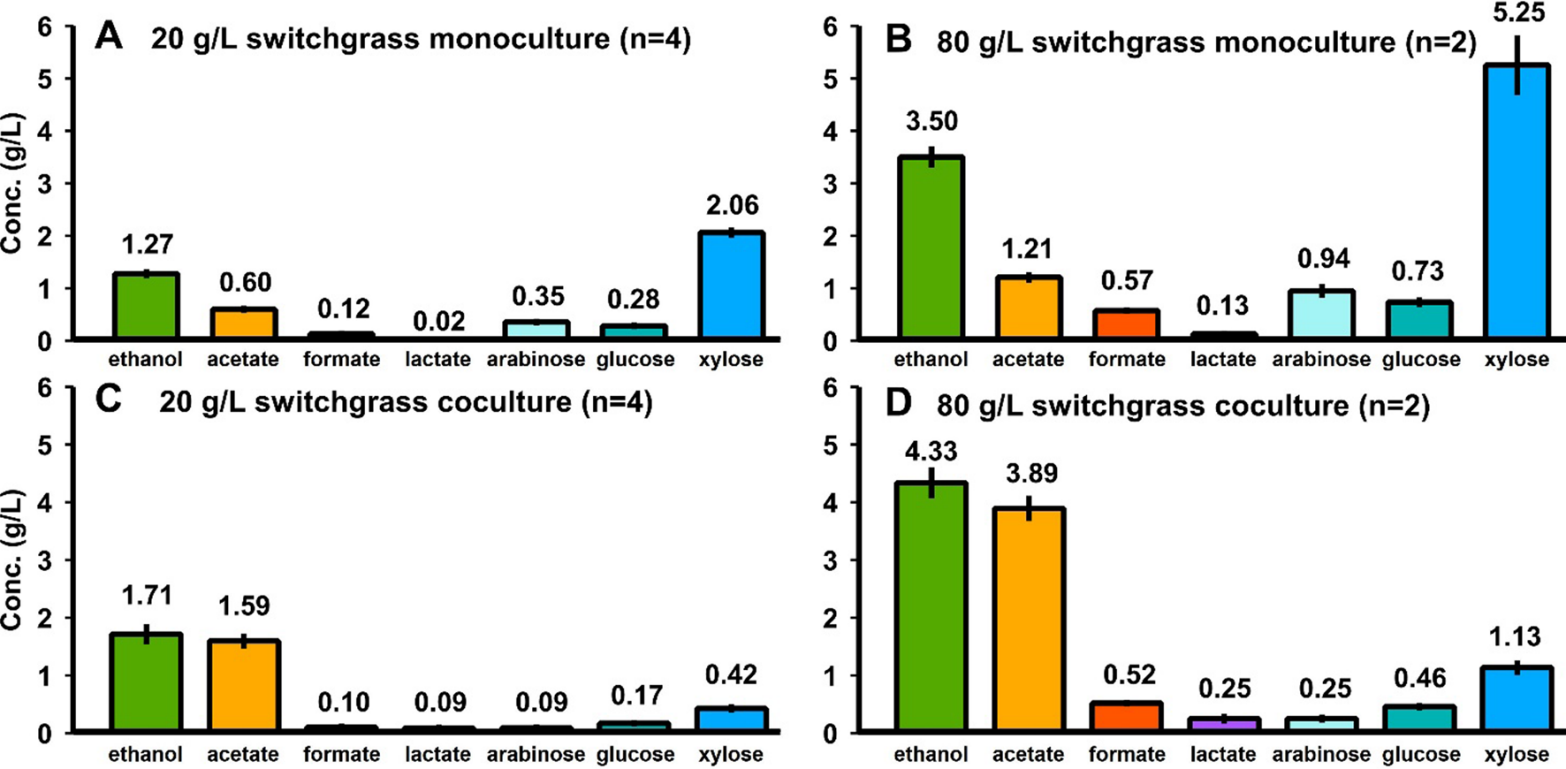
Monocultures and cocultures fermentation products and residual soluble carbohydrate for switchgrass. 7-day fermentations of senescent switchgrass by monocultures at loadings of 20 g/L (**A**) and 80 g/L (**B**) and cocultures at loadings of 20 g/L (**C**) and 80 g/L (**D**). Residual soluble carbohydrate was quantified in monomeric form after mild acid hydrolysis of the supernatant

Figures 7 and 8 illustrate the effect of adding *T. thermosaccharolyticum* as a hemicellulose-utilizing coculture partner to cultures of *C. thermocellum*. Under the chosen conditions, the coculture partner fermented most but not all of the soluble pentose-rich oligosaccharides made available by *C. thermocellum*-mediated lignocellulose deconstruction. Based on carbohydrate solubilization and feedstock acetyl content [55, 56], the fraction of acetate that was fermentative product for monocultures and cocultures on corn stover ranged between 77.4–77.7% and 82.0–83.9%, respectively. Similarly, for switchgrass, values ranged between 56.4–77.0% and 79.5–90.6%, respectively (Additional file 1: Table S10). Fractional carbohydrate solubilization and utilization data are presented in Table 1 for the experiments depicted in Figs. 5, 6, 7, 8.

**Table 1:** Comparison of carbohydrate solubilization and utilization for mono- and cocultures

	Solid loading	Fractional carbohydrate solubilization	Fractional utilization of solubilized glucose	Fractional utilization of solubilized xylose
Corn stover				
Monoculture	20 g/L	0.656 ± 0.017	0.938 ± 0.002	–
	80 g/L	0.557 ± 0.010	0.946 ± 0.001	–
Coculture	20 g/L	0.749 ± 0.014	0.957 ± 0.001	0.779 ± 0.027
	80 g/L	0.632 ± 0.002	0.958 ± 0.001	0.798 ± 0.002
Switchgrass				
Monoculture	20 g/L	0.406 ± 0.028	0.904 ± 0.015	–
	80 g/L	0.259 ± 0.026	0.910 ± 0.003	–
Coculture	20 g/L	0.457 ± 0.042	0.952 ± 0.002	0.817 ± 0.002
	80 g/L	0.308 ± 0.021	0.954 ± 0.000	0.821 ± 0.002

Fractional sugar utilization calculated as one minus the mass of sugars found in the fermentation broth over the theoretical mass of sugars that have been solubilized based on fermented and unfermented solids. Sugar concentrations in the fermentation broth quantified in monomeric form via mild acid hydrolysis of the supernatant. No fractional utilization of xylose was detected in the monocultures

It may be observed that fractional carbohydrate solubilization is sensitive to solid loading, whereas the fractional utilization of solubilized carbohydrates is not. For cocultures on either feedstock, roughly 5% and 20% of the solubilized glucose- and xylose-oligomers, respectively, are not utilized.

**Fig. 9:**
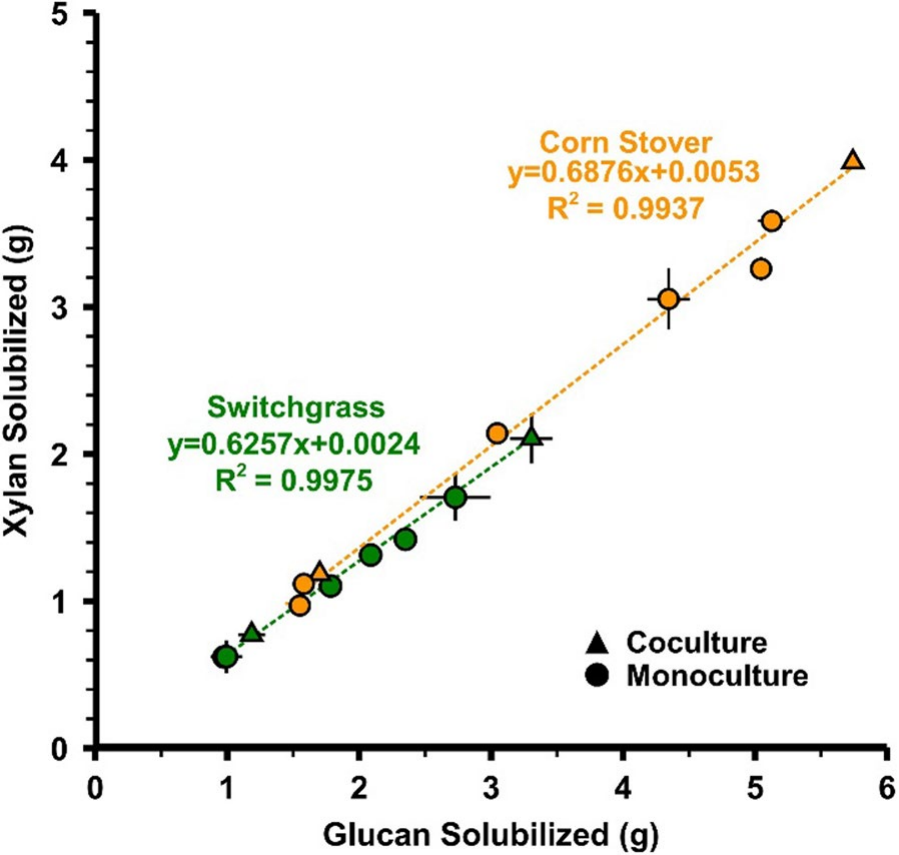
Linear solubilization ratio between xylan and glucan for different cultivation conditions. Datapoints are averages of the duplicate fermentations depicted in Fig. 1 and Fig. 5 and error bars represent 1 standard deviation. Trendlines are calculated using combined datasets for each feedstock which represent two culture types (mono- and cocultures) and multiple solid loadings (20, 40, 60, 80 g/L)

A linear relationship was observed for fractional solubilization of xylan and glucan for monocultures and cocultures fermenting both corn stover and switchgrass at various substrate loadings (Fig. 9). This relationship is largely maintained across solid loadings and culture type. This would suggest that solid loading and culture type does not bias the solubilization activity either towards or away from glucose (or xylose).

## Discussion

Here, we document declining solubilization with increasing solids loading over a range of 20 to 80 g/L for batch monocultures of *C. thermocellum* fermenting either corn stover or senescent switchgrass. Our observation of this trend is consistent with results of both Verbeke et al. and Shao et al. on mid-season switchgrass at various solids loadings [19, 52]. Comparing monocultures with 80 g/L and 20 g/L substrate loadings with no pretreatment other than autoclaving, final total carbohydrate solubilization declined from 0.695 to 0.565 for corn stover and from 0.399 to 0.243 for switchgrass. Data at intermediate solids loading indicate a roughly linear declining solubilization trend for both substrates. Remediation of this effect is likely necessary for commercial application acknowledging that substrate loadings about twofold higher than the maximum studied herein are generally envisioned for economically viable production of ethanol from lignocellulose [2, 3]. Such remediation would be greatly informed, and likely require, understanding the factor(s) responsible for the declining fractional solubilization trend observed here.

As reported previously, we routinely observe near-complete utilization of Avicel-cellulose up to 120 g/L by *C. thermocellum* cultures [49–51], which is accompanied by concentrations of fermentation products over twice those observed herein with corn stover or switchgrass at 80 g/L. In light of these results, both inhibition by fermentation products and limitation by inadequate amounts of growth medium components seem unlikely to explain the declining fractional solubilization trend. Several alternative hypotheses for the basis of this phenomenon were tested but did not confirm specific factors. Accumulation of liquid-phase inhibitors is a common explanation for cellulase inhibition, yet in this study, *C. thermocellum* was able to overcome a transient delay in cellulose utilization in the presence of spent broth from high lignocellulose loadings. Addition of spent broth from fermentation of 20 and 80 g/L corn stover and switchgrass (Fig. 3) resulted in little to no impact on cellobiose fermentation, indicating that fermentation of glucan solubilization products was not inhibited, and little if any long-term impact on the generation of fermentation products from cellulose, suggesting that cellulase activity is not irreversibly inhibited. In this respect, our results differ from those of Beri et al. who observed inhibition from corn fiber spent broth on cellobiose incubations by *C. thermocellum* monocultures [20]. It should be noted that there are several compounding factors that obscure comparisons between spent broth experiments, including cultivation conditions and spent broth preparation, and similar results for corn stover and corn fiber would not necessarily be expected considering the markedly different feedstock composition and experimental design [20, 28]. For cellulose incubations in the presence of switchgrass spent broth, Verbeke et al. observed reductions in solubilization for 17.5 g/L cellulose, but showed little difference in net-end product concentrations, as we also observed [52]. Shao et al. (2020) observed decreased cellulose conversion due to added spent broth measured at 24 h, however measurements were not taken at longer times to determine if conversion with added broth eventually equaled the controls as was observed in Fig. 3 [19].

Mid-fermentation addition of cellobiose or cellulose to 80 g/L corn stover *C. thermocellum* monocultures resulted in a dramatic increase in gas formation compared to controls without substrate added, but no significant increase in FCS. This suggests that the culture and its cellulases were active and not limited in utilizing additional (model) substrate, but rather had stopped solubilizing additional substrate from lignocellulose. Note that the additional utilization of cellobiose and cellulose could have resulted in an increase in cellular biomass or posed an opportunity for new cellular biomass to be generated. Furthermore, no significant increase in solubilization was observed for additions of biocatalyst (concentrated cells) suggesting limitations in solubilization could be more closely related to the lignocellulosic substrate rather than *C. thermocellum’s* enzymatic machinery. An increase in FCS was observed for an addition of 60 ml water to a 300-ml culture which effectively diluted the initial substrate loading at 80 to 66.7 g/L. Linear interpolation of carbohydrate solubilization at 60 and 80 g/L from Fig. 1 gives an expected solubilization of 0.606 at a substrate loading of 66.7 g/L, which is very close to the observed value of 0.619. Thus, results with different initial substrate loadings are very nearly recapitulated by a water dilution during active fermentation.

Coculturing *C. thermocellum* with the hemicellulose-fermenting *Thermoanaerobacterium thermosaccharolyticum* increased solubilization compared to monocultures at both 20 g/L and 80 g/L substrate loadings for corn stover (*p* < 0.001) and switchgrass (*p* = 0.063). Inclusion of *T. thermosaccharolyticum* as a coculture partner did not, however, ameliorate declining solubilization as was observed at 40 g/L corn fiber by Beri et al. (from 67 to 93%) although the absolute amount of carbohydrates solubilized and utilized is much higher for corn stover [20]. For each feedstock, we observed a consistent fraction of hemicellulose sugars are being solubilized but not utilized regardless of solid loadings (Table 2). Our results are consistent with certain linkages present in soluble products of *Clostridium thermocellum*-mediated hemicellulose deconstruction being inaccessible to the array of carbohydrate active enzymes of *T. thermosaccharolyticum,* as had been previously observed by Beri et al. for corn fiber [28].

**Table 2:** Lignocellulose feedstock characteristics of 60 °C oven-dried unfermented feedstock

Feedstock	Carbohydrates (g/g)			Moisture (wt.%)	Ash (wt.%)	Lignin (wt.%)
	Glucose	Xylose	Arabinose			
POET corn stover A	0.366 ± 0.005	0.274 ± 0.005	0.032 ± 0.000	1.310 ± 0.509	13.010 ± 0.000	18.333 ± 0.005
POET corn stover B	0.417 ± 0.002	0.301 ± 0.000	0.0405 ± 0.000	1.165 ± 0.176	6.769 ± 0.000	16.191 ± 0.001
ERNST senescent switchgrass	0.435 ± 0.000	0.298 ± 0.000	0.029 ± 0.000	1.170 ± 0.339	2.961 ± 0.000	19.483 ± 0.002

To be consistent with the observations reported herein, a proposed mechanism for declining fractional carbohydrate solubilization with increasing solids would need to be lignocellulose-specific, involve factors other than nutrient limitation and inhibition by liquid-phase products of fermentation or deconstruction, not be due to inactive microbial cells or CAZymes, be reversed by liquid-phase dilution, and partially reversed by addition of a hemicellulose-fermenting coculture partner. In general, a mechanism involving decreasing lignocellulose accessibility with increasing substrate loading seems mostly likely to us. It has been previously reported that xylan and pentose-rich oligosaccharides adsorb onto cellulose surfaces [37, 58–61], and cellulase performance is generally enhanced in the presence of hemicellulolytic enzymes [37–40]. Noting that dilution of the liquid phase would be expected to cause adsorbed oligosaccharides to enter solution by Le Chatelier’s principle, impediment of deconstruction by adsorbed oligosaccharides would appear to be a plausible mechanism for *C. thermocellum* which remains to be definitively proven.

## Conclusions

Decreasing fractional carbohydrate solubilization with increasing substrate loading was observed for monocultures of *C. thermocellum* and coculture fermentations of *C. thermocellum* and *T. thermosaccharolyticum* fermenting corn stover and senescent switchgrass with no pretreatment other than autoclaving. Results of experiments involving spent media addition do not support a major role for inhibitors present in the liquid phase. Substrate addition experiments confirm that *C. thermocellum* and its CAZymes remain capable of converting model substrates during the middle of lignocellulose fermentation at the maximum substrate loading tested (80 g/L). Substrate dilution reverses the observed decrease in fractional carbohydrate solubilization at increasing substrate loading, and coculture of *C. thermocellum* with *T. thermosaccharolyticum*, which ferments hemicellulose, increases fractional carbohydrate solubilization compared to *C. thermocellum* monocultures at both lower (20 g/L) and higher (80 g/L) loadings of both corn stover and switchgrass. For both monocultures and cocultures, regardless of solid loading, there remains a consistent fraction of carbohydrates that undergo solubilization, but not utilization. This suggests that some carbohydrates are unaffected by the existing suite of CAZymes present in the culture. Impediment of deconstruction by adsorbed oligosaccharides would appear to be a plausible mechanism for the observed trend of declining solubilization with increasing substrate loading, although this remains to be conclusively shown.

## Methods

### Substrates

Carbohydrate solubilization of two lignocellulosic feedstocks were characterized across various solid loadings. Corn stover was a gift from POET Research Inc (Sioux Falls, SD) pre-milled at 1/8″ size material. Due to material limitations, a separate batch of corn stover (also from POET Research Inc) was used for experiments comparing monocultures and cocultures. Senescent lowland ‘Timber’ switchgrass was a gift from Ernst Seeds (Meadville PA, USA), planted in May 2016, harvested December 2018 and dried at 300–400˚F upon harvesting and milled at ¼” size for storage. Prior to characterization and use in experiments, both feedstocks were milled to pass through a 0.5-mm sieve on a Retsch ZM 200 centrifugal mill (Verder Scientific, Newton PA) [7]. Carbohydrate content was determined by quantitative saccharification (QS) with 72% (w/w) H_2_SO_4_ (Fisher, Waltham MA), as described in Sluiter et al. [62]. Post-QS-sample supernatant was filtered via Spin-X centrifuge tubes (0.22 µm nylon) (Corning, Corning NY) before HPLC analysis. The acid-hydrolyzed monomeric sugars arabinose, glucose, and xylose were quantified by refractive index detection and separated via HPLC (Waters, Milford MA) with an Aminex® HPX-87H column (Bio-Rad, Hercules CA) operating at 60 ℃, 2.5 mM H_2_SO_4_ eluent, at a flowrate of 0.6 mL/min [7]. Moisture content was determined using an A&D MX-50 Moisture Analyzer (San Jose, CA). Ash content was determined according to the method described in Sluiter et al. [63] using a Dentsply-Ceramco Vulcan 3–550 muffle furnace (Dentsply-Sirona, York, PA) (Ceramco, Conway, NH). Lignin content was approximated as 100% minus the sum of carbohydrates, moisture, and ash content [63]. Each value recorded in Table 2 was determined using feedstocks dried at 60 °C, whereas fractional carbohydrate solubilization was calculated using air-dried, room temperature unfermented feedstock. Average carbohydrate content was determined in triplicate and moisture, ash, and lignin in duplicate.

### Microbial strains and growth media

*Clostridium thermocellum* DSM1313 (LL1004) was obtained from the Deutsche Sammlung von Mikroorganismen und Zellkulturen GmbH (DSMZ, Liebniz, Ger.). *Thermoanaerobacterium thermosaccharolyticum* HG-8 ATCC 31960 (LL1244) was obtained from the American Type Culture Collection (ATCC, Manassas, VA). Inocula were prepared by culturing *C. thermocellum* on MTC [64] and 50 g/L microcrystalline cellulose (Avicel® PH105, FMC biopolymers, Philadelphia PA) in a pH-controlled 1.2-L Sartorius bioreactor. During mid-growth phase, 30-mL cell culture aliquots were transferred to 50-mL serum bottles. Cell culture aliquots were stored at -80˚C and were slowly thawed several hours before bioreactor inoculation. *T. thermosaccharolyticum* inoculum was cultured on modified CTFüD medium substituted with D-xylose for cellobiose [65]. Inoculum was re-seeded several hours prior to bioreactor inoculation in order to ensure culture viability, which was added to bioreactors at 4%(v/v). Defined Medium for Thermophilic Clostridia (MTC) was prepared as described prior [64]; solution B had bioreactor concentrations of 2.12 g/L potassium citrate monohydrate (C_6_H_7_O_8_K_3_), 1.25 g/L citric acid monohydrate (C_6_H_8_O_7_ · H_2_O), 1.0 g/L Na_2_SO_4_, 1 g/L KH_2_PO_4_, 2.5 g/L NaHCO_3_, and was added at 4%(v/v). Solution C had a bioreactor concentration of 2.0 g/L urea (CH_4_N_2_O) and was added at 2%(v/v). Solution D had bioreactor concentrations of 1.0 g/L MgCl_2_ · 6H_2_O, 0.2 g/L CaCl_2_, 0.1 g/L FeCl_2_ · 4H_2_O, 1.0 g/L L-cysteine HCL monohydrate (C_3_H_7_NO_2_S · HCl · H_2_O), and was added at 2%(v/v). Solution E had bioreactor concentrations of 0.02 g/L pyridoxamine dihydrochloride, 0.004 g/L 4-aminobenzoic acid, 0.002 g/L D-biotin, 0.002 g/L vitamin B_12_, and was added at 2%(v/v). Solution TE had bioreactor concentrations of 0.00625 g/L MnCl_2_ · 4H_2_O, 0.0025 g/L ZnCl_2_, 0.000625 g/L CoCl_2_ · 6H_2_O, 0.000625 g/L NiCl_2_ · 6H_2_O, 0.000625 g/L CuSO_4_ · 5H_2_O, 0.000625 g/L H_3_BO_3_, 0.000625 g/L Na_2_MoO_4_ · 2H_2_O, and was added at 0.5%(v/v). Each MTC solution was purged by 20 cycles of alternating N_2_ gas and Vacuum for 45 s each. After purging, solution D was autoclaved for 30 min on liquid cycle.

### Monoculture fermentations at various solid loadings

All lignocellulose fermentations were performed at 300 mL working volume in 0.5-L Sartorius Qplus bioreactors (Sartorius, Bohemia NY). Each solids loading was run at least in duplicate (*n* = 2). Lignocellulosic feedstocks were suspended in Milli-Q water (MilliporeSigma, Burlington MA) and autoclaved for 90 min on a liquid/slow exhaust cycle. Overnight, the bioreactor’s headspace was sparged with ‘ultra pure’ N_2_ gas (Airgas, White River Junction VT) while being stirred at 300 RPM at 55˚C. After sparging the bioreactors, media solutions were added to the lignocellulose slurry via a 0.2 μm polyethersulfone (PES) sterile syringe filter (Corning, Corning NY).

Prior to inoculation, the pH was controlled at 7.0 or 6.5 using a gel-filled pH probe (Mettler-Toledo, Billerica MA) and automatic addition of 4 N KOH (Fisher, Waltham MA) via a peristaltic pump in the Sartorius control tower. Online gas production was monitored with a milligas flow meter (Ritter, Hawthorne NY) filled with 0.5 N HCl. Total volumetric gas production data were recorded by the accompanying Rigamo software. *C. thermocellum* inoculum was added at 2% (v/v), and the fermentations proceeded for 168 h.

### Harvesting residual solids and determination of carbohydrate solubilization and fermentation products

After fermentation, the bioreactor contents were harvested as a whole and centrifuged for 15 min at 16,000 × *g* at 4 ºC on an Avanti J-26S XP centrifuge using rotor JA-10. Both the residual solids and supernatant were collected to determine their respective carbohydrate concentrations. Fractional carbohydrate solubilization is calculated as the difference between carbohydrate mass in the unfermented feedstock versus the residual solids. Carbohydrate content in the residual solids was determined via QS as described in the feedstock material section. Similarly, the carbohydrate content of the supernatant was determined using a mild-acid hydrolysis step named liquid quantitative saccharification (LQS) adjusted from Sluiter et al. [62]. Analysis of fermentation products (acetate, ethanol, formate, lactate) was performed by mixing 35 µL 10% (v/v) H_2_SO_4_ and 700 µL fermentation supernatant and allowing the solution to sit for > 5 min to denature proteins. The acidified solution was spun down in the microcentrifuge at 21,130 × g and the supernatant was filtered via Spin-X centrifuge tubes (0.22 μm nylon) (Corning, Corning NY). The filtrate was quantified for products via HPLC and quantified by comparison to standard solutions. Relative acetate contributions from non-fermentative lignocellulose deconstruction were estimated using carbohydrate solubilization values and feedstock acetyl content as determined by Kumar et al. [56] and Wyman et al. (55) (Additional file 1: Table S10).

### Bottle fermentations with lignocellulose fermentation spent media

Serum bottles (30 mL) were prepared by adding either D( +) cellobiose (MilliporeSigma, Burlington MA), or microcrystalline cellulose (Avicel® PH105, FMC biopolymers, Philadelphia PA) to MilliQ water, purged with nitrogen gas, and autoclaved for 30 min on a liquid cycle. Carbon substrates were concentrated such that the concentration prior to inoculation was either 5.0 g/L cellulose or cellobiose. The final volume (20 mL) consisted of 75% spent media (or sterile and anaerobic water for controls) and 25% fresh substrate and MTC media. To buffer pH, solution A was also included at a concentration, prior to inoculation, of 5.0 g/L morpholinopropane sulfonic acid (MOPS) sodium salt and was added at 2%(v/v). MTC was added to each bottle as described above. However, for bottle fermentations solutions A, B, and C were autoclaved for 30 min on liquid cycle, and not filter-sterilized.

Supernatant was collected from the high solids monoculture batch fermentations described above. Harvesting the residual solids, collecting and preparing the supernatant, and inoculating bottle fermentations were all performed on the same day. The liquid phase from 80 g/L corn stover, 20 g/L corn stover, 80 g/L switchgrass and 20 g/L switchgrass fermentations were used for bottle experiments. The supernatant was subject to a second centrifugation for 10 min at 50,000 × *g* on an Avanti J-26S XP centrifuge using a JA-25.50 rotor. The supernatant was then vacuum filtered with a glass fiber membrane prefilter (MilliporeSigma, Burlington MA) and then vacuum filtered with a 0.45-um nylon membrane filter (MilliporeSigma, Burlington MA). Lastly, the supernatant was added through a 0.2-um PES syringe sterile filter into an empty and autoclaved serum bottle and was aseptically purged for 20 cycles as previously described.

In addition to collecting lignocellulosic supernatant, supernatant from batch fermentations on model substrates, cellobiose and cellulose, were collected for bottle experiments. Monoculture batch fermentations of cellobiose and cellulose were performed as described above for 0.5 L final volume in 1.2-L Sartorius bioreactors. Both substrates were loaded at a bioreactor concentration of 12.1 g/L, which is approximately equal to the amount of glucan solubilized in an 80 g/L corn stover fermentation as determined in prior experiments. The model substrate cultures were harvested at 48 h which corresponded to approximately 24 h after base addition had stopped. Supernatant was collected and filtered in the same procedure described above for lignocellulosic supernatant.

A one-way release valve was used aseptically to equilibrate pressure before inoculation. Bottles, run in triplicate (*n* = 3), were inoculated 2% (v/v) with a – 80 ℃ bottle of *C. thermocellum* as described above. Bottles were cultivated at 55 ℃ in a shaking incubator and sampled for product formation every 24 h for five days, with net product formation equal to the product concentration at the timepoint of sampling, minus the product concentration at time zero. To serve as a control (*n* = 1), a bottle with 75% (v/v) water instead of supernatant was included for each experimental condition totaling three individual controls for cellulose with water and three for cellobiose with water.

### Monoculture fermentations with substrate and biocatalyst additions

Bioreactor fermentations at 80 g/L corn stover were prepared in the same manner described above. During growth phase, determined to be approximately *t* = 48 h by monitoring gas production, respective solutions were added to the fermentations. Based on prior experiments, 60 ml cellulose (*n* = 2) and cellobiose (*n* = 2) solutions were added at a concentration that effectively doubled the observed amount of glucan solubilized (5.524 g) in an uninterrupted 80 g/L corn stover fermentation. Biocatalyst was obtained by culturing *C. thermocellum* LL1004 on MTC containing 25 g/L cellobiose in a 1.2-L Sartorius bioreactor with a 1.0 L working volume. The fermentation broth was anaerobically and aseptically transferred out of the bioreactor during mid-growth phase (*t* = 24 h) by a peristaltic pump, and further processed in an anaerobic glovebag (Coy Laboratory Products, Grass Lakes, MI). The broth was aseptically transferred into centrifuge bottles and the cell mass pelleted at 16,000 × g for 5 min. The cell pellet was anaerobically resuspended in 200 mL (water), and 60 mL was immediately added to each bioreactor via syringe (*n* = 2). As a control, 60 ml of anaerobic and autoclaved MilliQ water was added at the same timepoint (*t* = 48 h) to an otherwise unaltered 80 g/L corn stover fermentations (*n* = 2). Bioreactors proceeded until *t* = 168 h, where they are harvested as described above.

### Coculture batch fermentations with low and high solids loadings

Two additional media solutions were added to support coculture batch fermentations of *C. thermocellum* and *T. thermosaccharolyticum.* First, a 2% (v/v) ammonium chloride (NH_4_Cl) solution was added with a bioreactor concentration of 2 g/L. Second, a 4% (v/v) vitamin solution was added with bioreactor concentrations of 0.004 g/L thiamine and 0.004 g/L thioctic acid. For experiments comparing monocultures and cocultures, these two additional solutions were also given to monocultures to ensure an identical media background. Additionally, a third solution of xylose-free CTFüD was added in place of *T. thermosaccharolyticm* inoculum (4% v/v) for monocultures. The xylose-free CTFüD is identical to the aforementioned CTFüD medium, except does not include the primary carbon substrate, D-xylose. This was done to control for the presence of yeast extract persisting in the *T. thermosaccharolyticum* inoculum in the coculture runs. Fractional utilization of solubilized carbohydrates was determined based on concentrations in the unfermented feedstock, fermented solids, and spent broth as determined by quantitative saccharification protocols. Fractional sugar utilization was calculated as one minus the mass of sugars found in the liquid phase ($m_{{\text{sugar, liquid phase}}} )$ over the theoretical mass of sugars that have been solubilized based on fermented and unfermented solids ($m_{{\text{sugar, unfermented solids}}} - m_{{\text{sugar, fermented solids}}} ).$

$$ {\text{Fractional sugar utilization}} = 1 - \frac{{m_{{\text{sugar, liquid phase}}} }}{{m_{{\text{sugar, unfermented solids}}} - m_{{\text{sugar, fermented solids}}} }}. $$

## Supplementary Information

**Additional file 1: Table S1**. Data from figure 1 and figure 2, fractional carbohydrate solubilization (FCS) and fermentation end product concentrations.** Table S2**. Molar product ratios for increasing solids.** Table S3**. Data from figure 2, residual solubilized carbohydrates.** Table S4**. Data from figure 3, fermentation end product ratios.** Figure S5**. Residual solubilized carbohydrates per addition.** Figure S6**. Fermentation products per addition.** Figure S7**. Xylose utilization by cocultures on defined medium.** Table S8**. Data from figure 5 and figure 7, fractional carbohydrates solubilization and fermentation end product and residual solubilized carbohydrate concentrations for corn stover fermentations.** Table S9**. Data from figure 5 and figure 8, fractional carbohydrates solubilization and fermentation end product and residual solubilized carbohydrate concentrations for senescent switchgrass fermentations.** Table S10**. Data from Figure 1, 2, 5, 7 and 8, estimated contributions towards reported acetate titers from either lignocellulose deconstruction or microbial fermentation product.

## Data Availability

All data generated and reported during this study are included in the published article and its additional files.

## Abbreviations

CBP: Consolidated bioprocessing
C-CBP: Consolidated bioprocessing with cotreatment
FCS: Fractional carbohydrate solubilization
AD: Anaerobic digestion
Av: Avicel
CB: Cellobiose
QS: Quantitative saccharification
LQS: Liquid quantitative saccharification
CBI: Center for Bioenergy Innovation
MTC: Media for thermophilic *Clostridia*
PES: Polyethersulfone
MOPS: Morpholinopropane sulfonic acid
CAZymes: Carbohydrate-active enzymes

## References

[1] Himmel ME, Ding S-Y, Johnson DK, Adney WS, Nimlos MR, Brady JW (2007). Biomass recalcitrance: engineering plants and enzymes for biofuels production. Science.

[2] Lynd LR, Liang X, Biddy MJ, Allee A, Cai H, Foust T, et al. Cellulosic ethanol: status and innovation. Vol. 45, Current opinion in biotechnology. Elsevier Ltd; 2017. p. 202–11. 10.1016/j.copbio.2017.03.008 28528086

[3] Humbird D, Davis R, Tao L, Kinchin C, Hsu D, Aden A, et al. Process design and economics for biochemical conversion of lignocellulosic biomass to ethanol: dilute-acid pretreatment and enzymatic hydrolysis of corn stover. 2002.

[4] Mosier N, Wyman C, Dale B, Elander R, Lee YY, Holtzapple M (2005). Features of promising technologies for pretreatment of lignocellulosic biomass. Biores Technol.

[5] Hu F, Ragauskas A (2012). Pretreatment and lignocellulosic chemistry. Bioenergy Res..

[6] Lynd LR, Weimer PJ, van Zyl WH, Pretorius IS (2002). Microbial cellulose utilization: fundamentals and biotechnology. Microbiol Mol Biol Rev.

[7] Paye JMD, Guseva A, Hammer SK, Gjersing E, Davis MF, Davison BH (2016). Biological lignocellulose solubilization: comparative evaluation of biocatalysts and enhancement via cotreatment. Biotechnol Biofuels.

[8] Balch ML, Holwerda EK, Davis MF, Sykes RW, Happs RM, Kumar R (2017). Lignocellulose fermentation and residual solids characterization for senescent switchgrass fermentation by: *Clostridium thermocellum* in the presence and absence of continuous in situ ball-milling. Energy Environ Sci.

[9] Holwerda EK, Worthen RS, Kothari N, Lasky RC, Davison BH, Fu C (2019). Multiple levers for overcoming the recalcitrance of lignocellulosic biomass. Biotechnol Biofuels.

[10] Xu Q, Resch MG, Podkaminer K, Yang S, Baker JO, Donohoe BS (2016). Cell Biology: dramatic performance of *Clostridium thermocellum* explained by its wide range of cellulase modalities. Sci Adv.

[11] Izquierdo JA, Pattathil S, Guseva A, Hahn MG, Lynd LR (2014). Comparative analysis of the ability of *Clostridium clariflavum* strains and *Clostridium thermocellum* to utilize hemicellulose and unpretreated plant material. Biotechnol Biofuels.

[12] Xiong W, Reyes LH, Michener WE, Maness PC, Chou KJ (2018). Engineering cellulolytic bacterium *Clostridium thermocellum* to co-ferment cellulose- and hemicellulose-derived sugars simultaneously. Biotechnol Bioeng.

[13] Demain AL, Newcomb M, Wu JHD (2005). Cellulase, *Clostridia*, and ethanol. Microbiol Mol Biol Rev.

[14] Zhang J, Tang M, Viikari L (2012). Xylans inhibit enzymatic hydrolysis of lignocellulosic materials by cellulases. Biores Technol.

[15] Zhai R, Hu J, Saddler JN (2018). The inhibition of hemicellulosic sugars on cellulose hydrolysis are highly dependant on the cellulase productive binding, processivity, and substrate surface charges. Biores Technol.

[16] Qing Q, Yang B, Wyman CE (2010). Xylooligomers are strong inhibitors of cellulose hydrolysis by enzymes. Biores Technol.

[17] Xue S, Uppugundla N, Bowman MJ, Cavalier D, da Costa Sousa L, Dale BE (2015). Sugar loss and enzyme inhibition due to oligosaccharide accumulation during high solids-loading enzymatic hydrolysis. Biotechnol Biofuels.

[18] Verbeke TJ, Giannone RJ, Klingeman DM, Engle NL, Rydzak T, Guss AM (2017). Pentose sugars inhibit metabolism and increase expression of an AgrD-type cyclic pentapeptide in *Clostridium thermocellum*. Sci Rep.

[19] Shao X, Murphy SJ, Lynd LR (2020). Characterization of reduced carbohydrate solubilization during *Clostridium thermocellum* fermentation with high switchgrass concentrations. Biomass Bioenerg.

[20] Beri D, Herring CD, Blahova S, Poudel S, Giannone RJ, Hettich RL (2021). Coculture with hemicellulose-fermenting microbes reverses inhibition of corn fiber solubilization by *Clostridium thermocellum* at elevated solids loadings. Biotechnol Biofuels.

[21] Levin DB, Verbeke TJ, Munir R, Islam R, Ramachandran U, Lal S, et al. Omics approaches for designing biofuel producing cocultures for enhanced microbial conversion of lignocellulosic substrates. In: Direct microbial conversion of biomass to advanced biofuels. Elsevier; 2015. p. 335–63.

[22] Ng TK, Ben-Bassat A, Zeikus JG (1981). Ethanol production by thermophilic bacteria: fermentation of cellulosic substrates by cocultures of *Clostridium thermocellum* and *Clostridium thermohydrosulfuricum*. Appl Environ Microbiol.

[23] Saddler JN, Chan MKH (1984). Conversion of pretreated lignocellulosic substrates to ethanol by Clostridium thermocellum in mono- and co-culture with *Clostridium thermosaccharolyticum* and *Clostridium thermohydrosulphuricurn*. Can J Microbiol.

[24] Liu Y, Yu P, Song X, Qu Y (2008). Hydrogen production from cellulose by co-culture of *Clostridium thermocellum* JN4 and *Thermoanaerobacterium thermosaccharolyticum* GD17. Int J Hydrogen Energy.

[25] He Q, Hemme CL, Jiang H, He Z, Zhou J (2011). Mechanisms of enhanced cellulosic bioethanol fermentation by co-cultivation of *Clostridium* and *Thermoanaerobacter spp*. Biores Technol.

[26] Wang F, Wang M, Zhao Q, Niu K, Liu S, He D (2019). Exploring the relationship between *Clostridium thermocellum* JN4 and *Thermoanaerobacterium thermosaccharolyticum* GD17. Front Microbiol.

[27] Froese A, Schellenberg J, Sparling R (2019). Enhanced depolymerization and utilization of raw lignocellulosic material by co-cultures of *Ruminiclostridium thermocellum* with hemicellulose-utilizing partners. Can J Microbiol.

[28] Beri D, York WS, Lynd LR, Peña MJ, Herring CD (2020). Development of a thermophilic coculture for corn fiber conversion to ethanol. Nat Commun.

[29] Kristensen JB, Felby C, Jørgensen H (2009). Yield-determining factors in high-solids enzymatic hydrolysis of lignocellulose. Biotechnol Biofuels.

[30] Weiss ND, Felby C, Thygesen LG (2019). Enzymatic hydrolysis is limited by biomass-water interactions at high-solids: improved performance through substrate modifications. Biotechnol Biofuels.

[31] Jørgensen H, Vibe-Pedersen J, Larsen J, Felby C (2007). Liquefaction of lignocellulose at high-solids concentrations. Biotechnol Bioeng.

[32] Mohagheghi A, Tucker M, Grohmann K, Wyman C (1992). High solids simultaneous saccharification and fermentation of pretreated wheat straw to ethanol. Appl Biochem Biotechnol.

[33] Hodge DB, Karim MN, Schell DJ, McMillan JD (2008). Soluble and insoluble solids contributions to high-solids enzymatic hydrolysis of lignocellulose. Biores Technol.

[34] Holtzapple M, Cognata M, Shu Y, Hendrickson C (1990). Inhibition of *Trichoderma reesei* celiulase by sugars and solvents. Biotechnol Bioeng.

[35] Jing X, Zhang X, Bao J (2009). Inhibition performance of lignocellulose degradation products on industrial cellulase enzymes during cellulose hydrolysis. Appl Biochem Biotechnol.

[36] Andrić P, Meyer AS, Jensen PA, Dam-Johansen K (2010). Reactor design for minimizing product inhibition during enzymatic lignocellulose hydrolysis: I. Significance and mechanism of cellobiose and glucose inhibition on cellulolytic enzymes. Biotechnol Adv.

[37] Malgas S, Kwanya Minghe VM, Pletschke BI (2020). The effect of hemicellulose on the binding and activity of cellobiohydrolase I, Cel7A, from *Trichoderma reesei* to cellulose. Cellulose.

[38] Qing Q, Wyman CE (2011). Supplementation with xylanase and β-xylosidase to reduce xylo-oligomer and xylan inhibition of enzymatic hydrolysis of cellulose and pretreated corn stover. Biotechnol Biofuels.

[39] Selig MJ, Knoshaug EP, Adney WS, Himmel ME, Decker SR (2008). Synergistic enhancement of cellobiohydrolase performance on pretreated corn stover by addition of xylanase and esterase activities. Biores Technol.

[40] Öhgren K, Bura R, Saddler J, Zacchi G (2007). Effect of hemicellulose and lignin removal on enzymatic hydrolysis of steam pretreated corn stover. Biores Technol.

[41] Berlin A, Balakshin M, Gilkes N, Kadla J, Maximenko V, Kubo S (2006). Inhibition of cellulase, xylanase and β-glucosidase activities by softwood lignin preparations. J Biotechnol.

[42] Rajan K, Carrier DJ (2014). Effect of dilute acid pretreatment conditions and washing on the production of inhibitors and on recovery of sugars during wheat straw enzymatic hydrolysis. Biomass Bioenerg.

[43] Wu G, Healy MG, Zhan X (2009). Effect of the solid content on anaerobic digestion of meat and bone meal. Biores Technol.

[44] Chen X, Yan W, Sheng K, Sanati M (2014). Comparison of high-solids to liquid anaerobic co-digestion of food waste and green waste. Biores Technol.

[45] Abbassi-Guendouz A, Brockmann D, Trably E, Dumas C, Delgenès JP, Steyer JP (2012). Total solids content drives high solid anaerobic digestion via mass transfer limitation. Biores Technol.

[46] Wang Z, Jiang Y, Wang S, Zhang Y, Hu Y, Hu Z (2020). Impact of total solids content on anaerobic co-digestion of pig manure and food waste: Insights into shifting of the methanogenic pathway. Waste Manag.

[47] Forster-Carneiro T, Pérez M, Romero LI (2008). Influence of total solid and inoculum contents on performance of anaerobic reactors treating food waste. Biores Technol.

[48] Fernández J, Pérez M, Romero LI (2008). Effect of substrate concentration on dry mesophilic anaerobic digestion of organic fraction of municipal solid waste (OFMSW). Biores Technol.

[49] Holwerda EK, Olson DG, Ruppertsberger NM, Stevenson DM, Murphy SJL, Maloney MI (2020). Metabolic and evolutionary responses of *Clostridium thermocellum* to genetic interventions aimed at improving ethanol production. Biotechnol Biofuels.

[50] Holwerda EK, Thorne PG, Olson DG, Amador-Noguez D, Engle NL, Tschaplinski TJ (2014). The exometabolome of *Clostridium thermocellum* reveals overflow metabolism at high cellulose loading. Biotechnol Biofuels.

[51] Argyros DA, Tripathi SA, Barrett TF, Rogers SR, Feinberg LF, Olson DG (2011). High ethanol Titers from cellulose by using metabolically engineered thermophilic, anaerobic microbes. Appl Environ Microbiol.

[52] Verbeke TJ, Garcia GM, Elkins JG (2017). The effect of switchgrass loadings on feedstock solubilization and biofuel production by *Clostridium thermocellum*. Biotechnol Biofuels.

[53] Basen M, Rhaesa AM, Kataeva I, Prybol CJ, Scott IM, Poole FL (2014). Degradation of high loads of crystalline cellulose and of unpretreated plant biomass by the thermophilic bacterium *Caldicellulosiruptor bescii*. Biores Technol.

[54] Straub CT, Khatibi PA, Otten JK, Adams MWW, Kelly RM (2019). Lignocellulose solubilization and conversion by extremely thermophilic *Caldicellulosiruptor bescii* improves by maintaining metabolic activity. Biotechnol Bioeng.

[55] Wyman CE, Balan V, Dale BE, Elander RT, Falls M, Hames B (2011). Comparative data on effects of leading pretreatments and enzyme loadings and formulations on sugar yields from different switchgrass sources. Biores Technol.

[56] Kumar R, Mago G, Balan V, Wyman CE (2009). Physical and chemical characterizations of corn stover and poplar solids resulting from leading pretreatment technologies. Biores Technol.

[57] Zhang Y-HP, Lynd LR (2005). Cellulose utilization by *Clostridium thermocellum*: bioenergetics and hydrolysis product assimilation. Proc Natl Acad Sci.

[58] Shi J, Ebrik MA, Yang B, Garlock RJ, Balan V, Dale BE (2011). Application of cellulase and hemicellulase to pure xylan, pure cellulose, and switchgrass solids from leading pretreatments. Biores Technol.

[59] Gu J, Catchmark JM (2013). The impact of cellulose structure on binding interactions with hemicellulose and pectin. Cellulose.

[60] Köhnke T, Östlund Å, Brelid H (2011). Adsorption of arabinoxylan on cellulosic surfaces: influence of degree of substitution and substitution pattern on adsorption characteristics. Biomacromol.

[61] Wang X, Li K, Yang M, Zhang J (2016). Hydrolyzability of xylan after adsorption on cellulose: exploration of xylan limitation on enzymatic hydrolysis of cellulose. Carbohyd Polym.

[62] Sluiter A, Hames B, Ruiz R, Scarlata C, Sluiter J, Templeton D, et al. Determination of structural carbohydrates and lignin in biomass: laboratory analytical procedure (LAP) (Revised July 2011). 2008.

[63] Sluiter A, Hames B, Ruiz R, Scarlata C, Sluiter J, Templeton D. Determination of ash in biomass: laboratory analytical procedure (LAP); Issue Date: 7/17/2005. 2008.

[64] Holwerda EK, Hirst KD, Lynd LR (2012). A defined growth medium with very low background carbon for culturing *Clostridium thermocellum*. J Ind Microbiol Biotechnol.

[65] Olson DG, Lynd LR. Transformation of *Clostridium thermocellum* by electroporation. In: Methods in enzymology. Academic Press Inc.; 2012. p. 317–30. 10.1016/B978-0-12-415931-0.00017-3 22608734

