# Supplementary material for: Declining carbohydrate solubilization with increasing solids loading during fermentation of cellulosic feedstocks by Clostridium thermocellum: documentation and diagnostic tests
Source: Biotechnol Biofuels Bioprod. 2022 Feb 5;15:12. doi: 10.1186/s13068-022-02110-4 (PMC8817502; doi:10.1186/s13068-022-02110-4)
Supplement: Supplementary file 1 — Additional file 1: Table S1. Data from figure 1 and figure 2, fractional carbohydrate solubilization (FCS) and fermentation end product concentrations. Table S2. Molar product ratios for increasing solids. Table S3. Data from figure 2, residual solubilized carbohydrates. Table S4. Data from figure 3, fermentation end product ratios. Figure S5. Residual solubilized carbohydrates per addition. Figure S6. Fermentation products per addition. Figure S7. Xylose utilization by cocultures on defined medium. Table S8. Data from figure 5 and figure 7, fractional carbohydrates solubilization and fermentation end product and residual solubilized carbohydrate concentrations for corn stover fermentations. Table S9. Data from figure 5 and figure 8, fractional carbohydrates solubilization and fermentation end product and residual solubilized carbohydrate concentrations for senescent switchgrass fermentations. Table S10. Data from Figure 1, 2, 5, 7 and 8, estimated contributions towards reported acetate titers from either lignocellulose deconstruction or microbial fermentation product. [file 13068_2022_2110_MOESM1_ESM.docx]

**Additional File 1. Primary and Supplemental Data**

S1: Table S1. Data from figure 1 and figure 2, fractional carbohydrate solubilization (FCS) and fermentation end product concentrations.

| (n=2) | Corn Stover | | | | | Senescent Switchgrass | | | | |
| --- | --- | --- | --- | --- | --- | --- | --- | --- | --- | --- |
| Loading | FCS | Ethanol (g/L) | Acetate (g/L) | Formate (g/L) | Lactate (g/L) | FCS | Ethanol (g/L) | Acetate (g/L) | Formate (g/L) | Lactate (g/L) |
| 20 g/L | 0.695  ±0.013 | 0.962 ±0.007 | 1.634 ±0.097 | 0.213 ±0.028 | 0.063 ±0.013 | 0.399 ±0.001 | 0.658  ±0.006 | 1.286 ±0.018 | 0.108 ±0.001 | 0.020 ±0.001 |
| 40 g/L | 0.663 ±0.006 | 1.657 ±0.028 | 2.953 ±0.169 | 0.299 ±0.025 | 0.188 ±0.008 | 0.365 ±0.009 | 1.048 ±0.070 | 2.053 ±0.088 | 0.063 ± ±0.003 | 0.027 ±0.003 |
| 60 g/L | 0.630 ±0.033 | 2.087 ±0.085 | 3.630 ±0.029 | 0.428 ±0.021 | 0.207 ±0.000 | 0.291 ±0.007 | 1.136 ±0.046 | 2.732 ±0.082 | 0.230 ±0.007 | 0.036 ±0.000 |
| 80 g/L | 0.556 ±0.011 | 2.655 ± ±0.028 | 5.071 ±0.224 | 0.767 ±0.056 | 0.359 ±0.010 | 0.2436 ±0.005 | 1.202 ±0.031 | 3.526 ±0.140 | 0.300 ±0.002 | 0.040 ±0.005 |

S2: Table S2. Molar product ratios for increasing solids

| Molar Product Ratios | | | | | | | | |
| --- | --- | --- | --- | --- | --- | --- | --- | --- |
| *(n=2)* | Corn Stover | | | | Senescent Switchgrass | | | |
| Loading | Ethanol | Acetate | Formate | Lactate | Ethanol | Acetate | Formate | Lactate |
| 20 g/L | 1.000 | 1.303 | 0.277 | 0.033 | 1.000 | 1.499 | 0.168 | 0.016 |
| 40 g/L | 1.000 | 1.201 | 0.185 | 0.036 | 1.000 | 1.503 | 0.159 | 0.013 |
| 60 g/L | 1.000 | 1.334 | 0.210 | 0.051 | 1.000 | 1.845 | 0.207 | 0.016 |
| 80 g/L | 1.000 | 1.465 | 0.148 | 0.069 | 1.000 | 2.250 | 0.255 | 0.017 |

S3: Table S3. Data from figure 2, residual solubilized carbohydrates.

| (n=2) | Corn Stover | | | Senescent Switchgrass | | |
| --- | --- | --- | --- | --- | --- | --- |
| Loading | Arabinose (g/L) | Glucose (g/L) | Xylose (g/L) | Arabinose (g/L) | Glucose (g/L) | Xylose (g/L) |
| 20 g/L | 0.704 ± 0.015 | 0.480 ± 0.018 | 2.899 ±0.172 | 0.322 ±0.013 | 0.279 ±0.007 | 1.680 ±0.054 |
| 40 g/L | 1.034 ±0.050 | 0.677 ±0.020 | 6.292 ±0.357 | 0.590 ±0.031 | 0.527 ±0.022 | 3.102 ±0.033 |
| 60 g/L | 1.456 ±0.054 | 0.931 ±0.041 | 8.778 ±0.298 | 0.751 ±0.003 | 0.649 ±0.000 | 3.781 ±0.033 |
| 80 g/L | 2.448 ± 0.000 | 1.192 ±0.035 | 10.157 ± 0.087 | 0.902 ±0.034 | 0.791 ±0.030 | 4.318 ±0.120 |

S4: Table S4. Data from figure 3, fermentation end product ratios.

| Spent Media Experiments Net Product Ratios | | | | | |
| --- | --- | --- | --- | --- | --- |
| Substrate | Spent Media | Ethanol (mol/L) | Acetate (mol/L) | Formate (mol/L) | Lactate (mol/L) |
| 5 g/L Avicel | Corn Stover 80 g/L | 1.000 | 1.101 | 0.070 | 0.089 |
| 5 g/L Avicel | Switchgrass 80 g/L | 1.000 | 0.897 | 0.271 | 0.044 |
| 5 g/L Avicel | Corn Stover 20 g/L | 1.000 | 1.158 | 0.083 | 0.016 |
| 5 g/L Avicel | Switchgrass 20 g/L | 1.000 | 0.863 | 0.293 | 0.028 |
| 5 g/L Avicel | Avicel 12.1 g/L | 1.000 | 0.732 | 0.322 | 0.218 |
| 5 g/L Avicel | Cellobiose 12.1 g/L | 1.000 | 0.997 | 0.335 | 0.019 |
| 5 g/L Avicel | water/none | 1.000 | 1.021 | 0.684 | 0.167 |
| 5 g/L Cellobiose | Corn Stover 80 g/L | 1.000 | 1.089 | 0.350 | 0.055 |
| 5 g/L Cellobiose | Switchgrass 80 g/L | 1.000 | 0.822 | 0.400 | 0.038 |
| 5 g/L Cellobiose | Corn Stover 20 g/L | 1.000 | 1.137 | 0.504 | 0.026 |
| 5 g/L Cellobiose | Switchgrass 20 g/L | 1.000 | 0.863 | 0.293 | 0.028 |
| 5 g/L Cellobiose | Avicel 12.1 g/L | 1.000 | 0.814 | 0.464 | 0.095 |
| 5 g/L Cellobiose | Cellobiose 12.1 g/L | 1.000 | 0.760 | 0.363 | 0.040 |
| 5 g/L Cellobiose | water/none | 1.000 | 0.887 | 0.449 | 0.095 |

S5: Figure S5. Residual solubilized carbohydrates per addition.


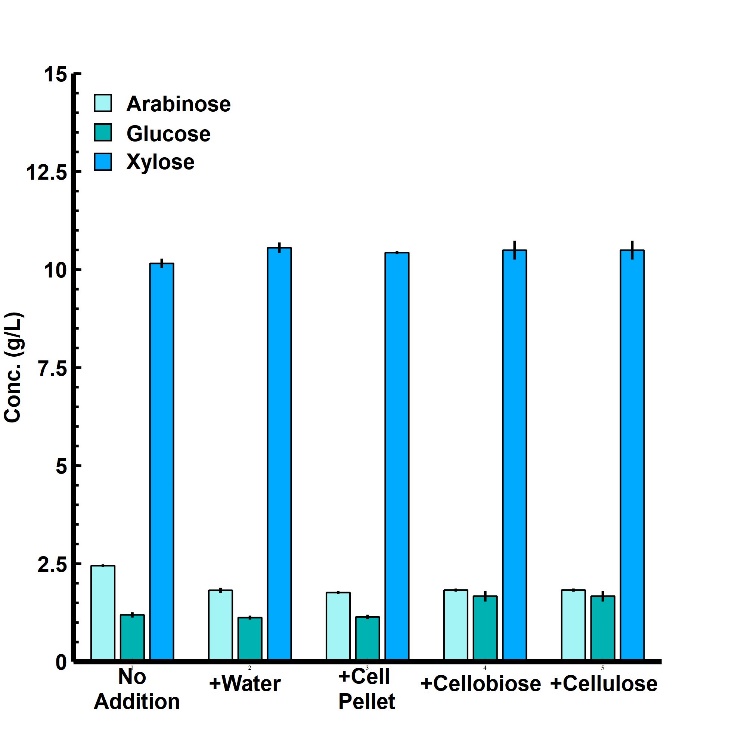


*Figure S5. Residual solubilized carbohydrate concentrations determined by mild acid hydrolysis of the supernatant for additions of model substrates and cells to 80 g/L corn stover fermentations. Concentrations are normalized to dilutions for comparison of results with and without additions. Error bars represent 1 standard deviation for duplicate bioreactor runs.*

Data Summary: This data emphasizes that additions of model substrates or cells did not improve xylose solubilization, which for monocultures of *C. thermocellum*, is a reasonable proxy for overall carbohydrate solubilization. Considering the overall carbohydrate solubilization did not significantly change (see figure 4) and the xylose concentrations are similar, it can be reasoned that the additional cellulose was nearly or completely consumed, and only a marginal increase in glucose (+0.15 and +0.54 g/L) was found in the supernatant for cellulose and cellobiose additions, respectively.

S6: Figure S6. Fermentation products per addition.


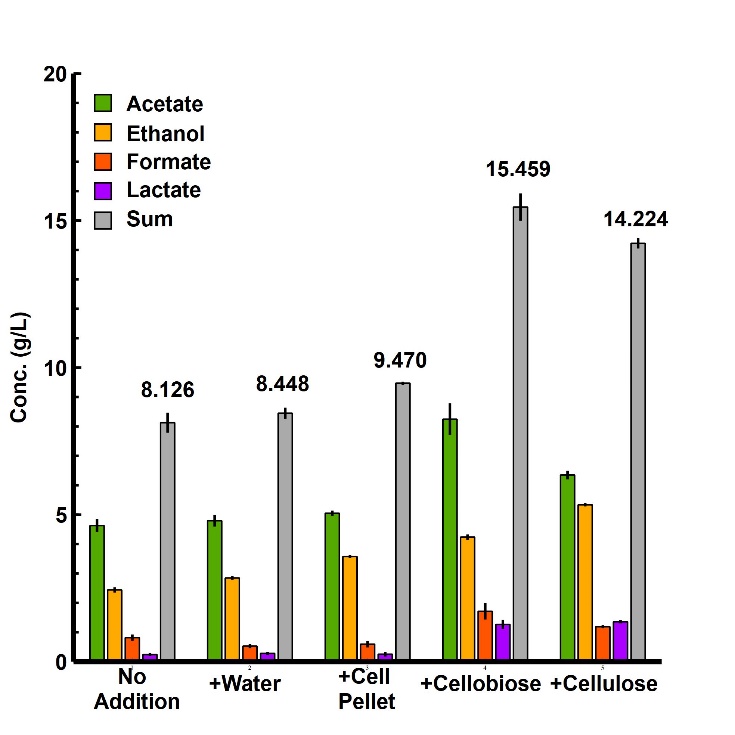


*Figure S6.* *Products ethanol, acetate*, formate, and lactate from fermentations of 80 g/L corn stover per addition. Sum refers to the addition of the above four product concentrations in g/L. Concentrations are normalized to dilutions for comparisons between no additions and additions. Error bars represent 1 standard deviation for duplicate bioreactor runs. *Acetate originates from both lignocellulose solubilization (cleavage of acetyl groups) and microbial fermentation of carbohydrates.*

Data Summary: Higher concentrations of fermentations products were observed for additions of cellobiose and cellulose. This data, along with gas production (figure 4B), suggests that the culture readily utilized the model substrates, and cellular limitations related to medium nutrients or product inhibition were not observed.

S7. Figure S7. Xylose utilization by cocultures on defined medium.


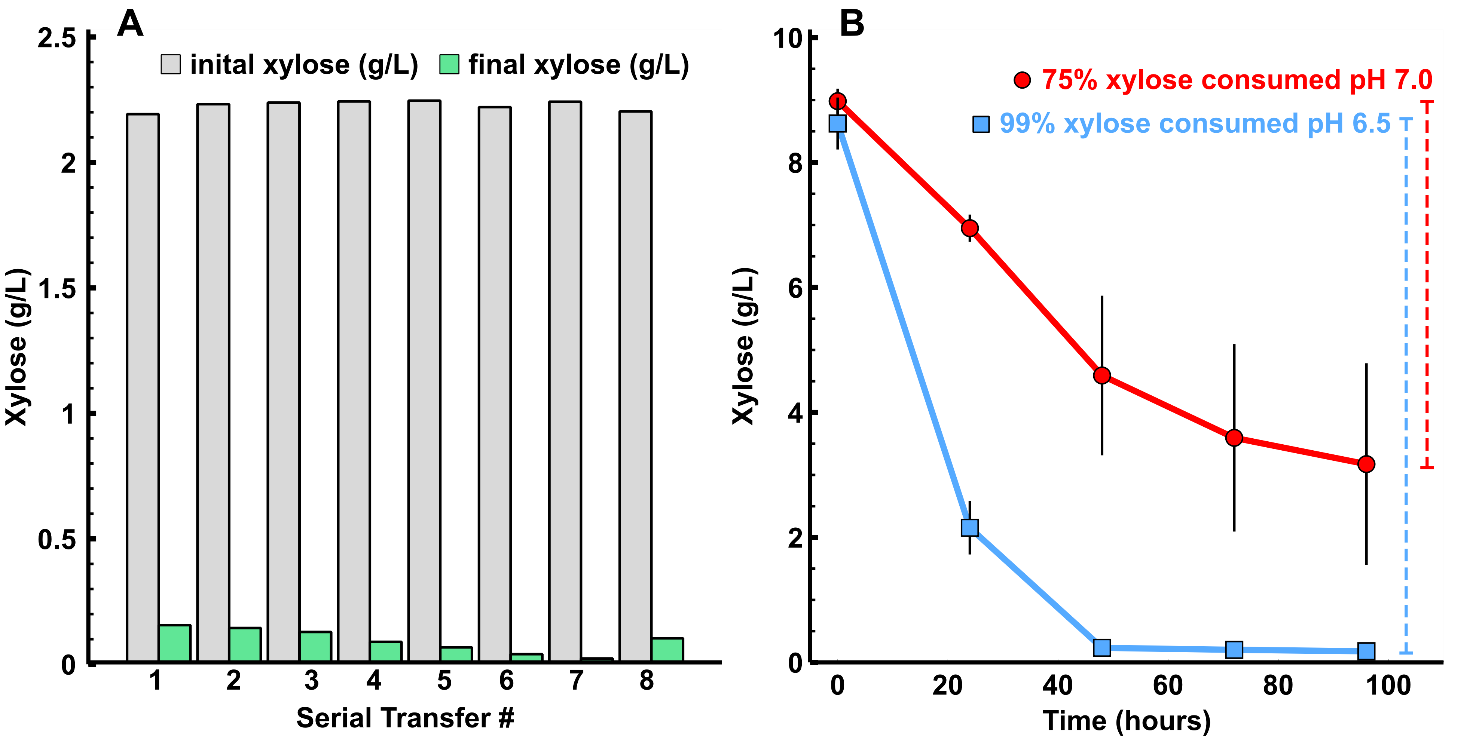


*Figure S7. Xylose utilization by cocultures. Panel (A) depicts initial (t=0 h) and final (t=24 h) xylose concentrations for a serially transferred coculture bottle fermentation. Defined MTC media with 2.5 g/L cellulose and 2.5 g/L xylose was pH buffered with 5 g/L working concentration of MOPS sodium salt. Panel (B) shows decreasing xylose concentrations across 24-hour samples for a coculture bioreactor fermentation. Defined MTC media with 20 g/L cellulose and 10 g/L xylose was pH controlled with additions of 4 N KOH. Concentration values are averages of duplicate bioreactor runs (n=2) and the error bars represent one standard deviation.*

Data Summary: Preliminary experiments demonstrate that pH is an influential factor in the fractional utilization of xylose by *T. thermosaccharolyticum* on defined media. Following experiments comparing monocultures and cocultures were operated at pH 6.5 to ensure reliable coculture partner performance.

Panel (A): It should be noted that the *T. thermosaccharolyticum* inoculum is prepared on undefined CTFUD medium containing yeast extract. The inoculum containing yeast extract that enters serial transfer bottle one is serially diluted (via 2%v/v serial inoculum) such that a negligible yeast extract concentration persists into the later serial transfers. Near-complete xylose utilization in the later serial transfers suggests that the coculture retains the ability to utilize xylose in the absence of yeast extract under defined pH-buffered MTC medium.

Panel (B): Under pH-controlled operating conditions (via additions of 4 N KOH), the coculture demonstrates near-complete utilization (~99%) of the xylose at pH 6.5 and unfinished xylose utilization (~75%) at pH 7.0. The variability is a great deal higher for the cocultures operating at pH 7.0, which could suggest that pH 7.0 is roughly the threshold for viable *T. thermosaccharolyticum* cultivations on xylose and defined medium.S8. Table S8. Data from figure 5 and figure 7, fractional carbohydrates solubilization and fermentation end product and residual solubilized carbohydrate concentrations for corn stover fermentations.

|  | Corn Stover | | | | | | | | |
| --- | --- | --- | --- | --- | --- | --- | --- | --- | --- |
| Culture  Type | Solid Loading (g/L) | FCS | Ethanol (g/L) | Acetate (g/L) | Formate (g/L) | Lactate (g/L) | Arabinose (g/L) | Glucose (g/L) | Xylose (g/L) |
| MC | 20  (n=4) | 0.655 ±0.016 | 0.932 ±0.031 | 1.483 ±0.078 | 0.188 ±0.004 | 0.033 ±0.003 | 0.530 ±0.006 | 0.290 ±0.006 | 3.293 ±0.083 |
| CC | 20  (n=4) | 0.749 ±0.014 | 2.080 ±0.049 | 2.333 ±0.122 | 0.166 ±0.011 | 0.055 ±0.009 | 0.205 ±0.020 | 0.220 ±0.013 | 0.794 ±0.088 |
| MC | 80  (n=2) | 0.556 ±0.010 | 2.707 ±0.044 | 5.020 ±0.034 | 0.438 ±0.007 | 0.234 ±0.001 | 1.603 ±0.006 | 0.824 ±0.003 | 10.337 ±0.016 |
| CC | 80  (n=2) | 0.632±  0.002 | 7.232 ±0.284 | 7.079 ±0.127 | 0.710 ±0.008 | 1.309 ±0.105 | 0.660 ±0.020 | 0.734 ±0.022 | 2.438 ±0.016 |

S9. Table S9. Data from figure 5 and figure 8, fractional carbohydrates solubilization and fermentation end product and residual solubilized carbohydrate concentrations for senescent switchgrass fermentations.

|  | Senescent Switchgrass | | | | | | | | |
| --- | --- | --- | --- | --- | --- | --- | --- | --- | --- |
| Culture  Type | Solid Loading (g/L) | FCS | Ethanol (g/L) | Acetate (g/L) | Formate (g/L) | Lactate (g/L) | Arabinose (g/L) | Glucose (g/L) | Xylose (g/L) |
| MC | 20  (n=4) | 0.405 ±0.028 | 0.597 ±0.016 | 1.278 ±0.031 | 0.129 ±0.013 | 0.023 ±0.005 | 0.355 ±0.011 | 0.283 ±0.009 | 2.061 ±0.045 |
| CC | 20  (n=4) | 0.457 ±0.042 | 1.597 ±0.083 | 1.717 ±0.127 | 0.100 ±0.016 | 0.088 ±0.009 | 0.093 ±0.007 | 0.171 ±0.008 | 0.428 ±0.022 |
| MC | 80  (n=2) | 0.259 ±0.026 | 1.209 ±0.048 | 3.502 ±0.147 | 0.573 ±0.005 | 0.130 ±0.007 | 0.947 ±0.079 | 0.736 ±0.040 | 5.254 ±0.519 |
| CC | 80  (n=2) | 0.307 ±0.020 | 3.894 ±0.172 | 4.338 ±0.222 | 0.521 ±0.011 | 0.250 ±0.039 | 0.251 ±0.023 | 0.462 ±0.018 | 1.136 ±0.080 |

S10. Table S10. Data from Figure 1, 2, 5, 7 and 8, estimated contributions towards reported acetate titers from either lignocellulose deconstruction or microbial fermentation product.

| Feedstock | Culture | pH | Lignocellulose Loading | | | FCS | Acetyl Content [1][2] | Theoretical Acetate Solubilized | | Total Acetate Titer | | |
| --- | --- | --- | --- | --- | --- | --- | --- | --- | --- | --- | --- | --- |
|  |  |  | (g) | (L) | (g/L) |  | (dry wt. %) | (g) | (g/L) | (g/L) | (% lignocellulose) | (% microbial) |
| corn stover | MC | 7 | 6 | 0.3 | 20 | 0.695 | 2.51% | 0.105 | 0.349 | 1.63 | 21.41% | 78.59% |
| corn stover | MC | 7 | 12 | 0.3 | 40 | 0.663 | 2.51% | 0.200 | 0.666 | 2.59 | 25.71% | 74.29% |
| corn stover | MC | 7 | 18 | 0.3 | 60 | 0.63 | 2.51% | 0.285 | 0.949 | 3.63 | 26.15% | 73.85% |
| corn stover | MC | 7 | 24 | 0.3 | 80 | 0.556 | 2.51% | 0.335 | 1.117 | 5.07 | 22.03% | 77.97% |
| switchgrass | MC | 7 | 6 | 0.3 | 20 | 0.399 | 3.60% | 0.086 | 0.287 | 1.28 | 22.44% | 77.56% |
| switchgrass | MC | 7 | 12 | 0.3 | 40 | 0.365 | 3.60% | 0.158 | 0.526 | 2.05 | 25.64% | 74.36% |
| switchgrass | MC | 7 | 18 | 0.3 | 60 | 0.291 | 3.60% | 0.189 | 0.629 | 2.73 | 23.02% | 76.98% |
| switchgrass | MC | 7 | 24 | 0.3 | 80 | 0.243 | 3.60% | 0.210 | 0.700 | 3.52 | 19.88% | 80.12% |
| corn stover | MC | 6.5 | 6 | 0.3 | 20 | 0.656 | 2.51% | 0.099 | 0.329 | 1.46 | 22.56% | 77.44% |
| corn stover | MC | 6.5 | 24 | 0.3 | 80 | 0.557 | 2.51% | 0.336 | 1.119 | 5.02 | 22.29% | 77.71% |
| corn stover | CC | 6.5 | 6 | 0.3 | 20 | 0.749 | 2.51% | 0.113 | 0.376 | 2.33 | 16.14% | 83.86% |
| corn stover | CC | 6.5 | 24 | 0.3 | 80 | 0.632 | 2.51% | 0.381 | 1.270 | 7.07 | 17.96% | 82.04% |
| switchgrass | MC | 6.5 | 6 | 0.3 | 20 | 0.406 | 3.60% | 0.088 | 0.292 | 1.27 | 23.02% | 76.98% |
| switchgrass | MC | 6.5 | 24 | 0.3 | 80 | 0.259 | 3.60% | 0.224 | 0.746 | 1.71 | 43.62% | 56.38% |
| switchgrass | CC | 6.5 | 6 | 0.3 | 20 | 0.457 | 3.60% | 0.099 | 0.329 | 3.5 | 9.40% | 90.60% |
| switchgrass | CC | 6.5 | 24 | 0.3 | 80 | 0.308 | 3.60% | 0.266 | 0.887 | 4.33 | 20.49% | 79.51% |

1. Kumar, R., Mago, G., Balan, V., & Wyman, C. E. (2009). Physical and chemical characterizations of corn stover and poplar solids resulting from leading pretreatment technologies. *Bioresource Technology*, *100*(17), 3948–3962. https://doi.org/10.1016/j.biortech.2009.01.075
2. Wyman, C. E., Balan, V., Dale, B. E., Elander, R. T., Falls, M., Hames, B., Holtzapple, M. T., Ladisch, M. R., Lee, Y. Y., Mosier, N., Pallapolu, V. R., Shi, J., Thomas, S. R., & Warner, R. E. (2011). Comparative data on effects of leading pretreatments and enzyme loadings and formulations on sugar yields from different switchgrass sources. *Bioresource Technology*, *102*(24), 11052–11062. https://doi.org/10.1016/j.biortech.2011.06.069
